# A Preliminary Metagenome Analysis Based on a Combination of Protein Domains

**DOI:** 10.3390/proteomes7020019

**Published:** 2019-04-29

**Authors:** Yoji Igarashi, Daisuke Mori, Susumu Mitsuyama, Kazutoshi Yoshitake, Hiroaki Ono, Tsuyoshi Watanabe, Yukiko Taniuchi, Tomoko Sakami, Akira Kuwata, Takanori Kobayashi, Yoshizumi Ishino, Shugo Watabe, Takashi Gojobori, Shuichi Asakawa

**Affiliations:** 1 Department of Aquatic Bioscience, Graduate School of Agricultural and Life Sciences, The University of Tokyo, Bunkyo, Tokyo 113-8657, Japan; aiga@mail.ecc.u-tokyo.ac.jp (Y.I.); dugongmanateewarlusseal@gmail.com (D.M.); akyoshita@g.ecc.u-tokyo.ac.jp (K.Y.); 2 Japan Software Management Co, Ltd., Yokohama, Kanagawa 221-0056, Japan; ono_h@jsm.co.jp; 3 Tohoku National Fisheries Research Institute, Japan Fisheries Research and Education Agency, Shiogama, Miyagi 985-0001, Japan; tsuyoshiw@affrc.go.jp (T.W.); taniuchi@affrc.go.jp (Y.T.); sakami@affrc.go.jp (T.S.); akuwata@affrc.go.jp (A.K.); 4 Hokkaido National Fisheries Research Institute, Japan Fisheries Research and Education Agency, Kushiro, Hokkaido 085-0802, Japan; 5 Research Center for Aquaculture Systems, National Research Institute of Aquaculture, Japan Fisheries Research and Education Agency, Minami-ise, Mie 516-0193, Japan; 6 National Research Institute of Fisheries Science, Japan Fisheries Research and Education Agency, Yokohama, Kanagawa 236-8648, Japan; kobayash@fra.affrc.go.jp; 7 Graduate School of Bioresorce and Bioenvironmental Sciences, Kyushu University, Fukuoka, Fukuoka 812-0053, Japan; ishino@agr.kyushu-u.ac.jp; 8 School of Marine Biosciences, Kitasato University, Sagamihara, Kanagawa 252-0373, Japan; swatabe@kitasato-u.ac.jp; 9 King Abdullah University of Science and Technology, Thuwal 23955, Saudi Arabia; takashi.gojobori@kaust.edu.sa

**Keywords:** protein domain, correlation coefficient, phylogenetic analysis, metagenomics, environmental DNA

## Abstract

Metagenomic data have mainly been addressed by showing the composition of organisms based on a small part of a well-examined genomic sequence, such as ribosomal RNA genes and mitochondrial DNAs. On the contrary, whole metagenomic data obtained by the shotgun sequence method have not often been fully analyzed through a homology search because the genomic data in databases for living organisms on earth are insufficient. In order to complement the results obtained through homology-search-based methods with shotgun metagenomes data, we focused on the composition of protein domains deduced from the sequences of genomes and metagenomes, and we utilized them in characterizing genomes and metagenomes, respectively. First, we compared the relationships based on similarities in the protein domain composition with the relationships based on sequence similarities. We searched for protein domains of 325 bacterial species produced using the Pfam database. Next, the correlation coefficients of protein domain compositions between every pair of bacteria were examined. Every pairwise genetic distance was also calculated from 16S rRNA or DNA gyrase subunit B. We compared the results of these methods and found a moderate correlation between them. Essentially, the same results were obtained when we used partial random 100 bp DNA sequences of the bacterial genomes, which simulated raw sequence data obtained from short-read next-generation sequences. Then, we applied the method for analyzing the actual environmental data obtained by shotgun sequencing. We found that the transition of the microbial phase occurred because the seasonal change in water temperature was shown by the method. These results showed the usability of the method in characterizing metagenomic data based on protein domain compositions.

## 1. Introduction

Approximately 71% of the Earth’s surface is covered by ocean, and 80% of life on this planet is believed to exist in this environment [1]. Furthermore, the marine environment, including the surface under the water, is thought to contain a total of approximately 3.67 × 10^30^ microorganisms, most of which are difficult to cultivate [2,3]. It has been twenty years since the advent of the word “metagenome” [4]. In the metagenomic analysis of marine bacterial communities, Venter et al., 2004, have conducted a metagenomic analysis in the Sargasso Sea [5]. As a result of this study, it has become widely accepted that approaches such as these can lead to the analysis of microbial communities in the environment and the discovery of novel useful genes. In recent years, global ocean metagenomic data have been gathered, such as in the Tara-Oceans project [6,7]. For downstream analysis, including gene prediction, it is important to prepare contigs with a high degree of continuity.

However, it is difficult to obtain a high-quality contig from a short-reads output by Illumina Hi-Seq and others, and the overlapping regions among different bacterial species make it even more difficult to assemble for metagenome analysis [8]. In addition, in the metagenomic analysis that uses current homology searching methods, there are cases where a large number of short reads cannot obtain useful information at all [9]. Although, in the case of protein domain information, there is a possibility that many short reads can cover functional regions called one or more protein domains. Such protein domains serve as important clues for the consideration of protein function. Pfam is one of the most well-maintained protein domain databases, and it holds a collection of an enormous number of protein families, the number of which continues to increase [10,11]. Its biggest attribute is that it uses a program called HMMER as a retrieval system, which allows for a fast and accurate search of the protein domains. In addition, it is expected that bacteria that are close to lineage similarly retain the protein domains, which means that they can be used as an element for characterizing the environment.

The combinations of protein genes in evolutionally close species are expected to show higher similarities [12,13]. Therefore, the similarity of combinations of protein domains among evolutionally close species is expected [14]. In this research, we carried out a metagenomic analysis using the information from the protein domains. We drew phylogenetic trees for combinations of protein domains, and we used 325 bacteria species that are found in previous studies [15] as the test samples, see Supplementary Table S1. DNA sequences of 16S ribosomal RNA (rRNA) [16], as well as DNA and amino acid sequences of the DNA gyrase subunit before comparison [17], using 367 bacteria species, were studied, as shown in the Supplementary Table S1. Next, DNA fragments were randomly extracted from the genomes of 325 bacteria of the same species and a phylogenetic tree was drawn for purposes of comparison. If our hypothesis is correct, these phylogenetic trees should be similar to some extent. In addition to the phylogenetic tree, the pairwise genetic distances between the domain combinations and the DNA or amino acid sequence were plotted and the methods were evaluated using the correlation coefficient. Finally, the method that was examined was applied to metagenomic data of actual environmental DNA and it was evaluated.

## 2. Materials and Methods

### 2.1. Creating Cluster Dendrograms for Combinations of Protein Domains

Throughout this paper, bacteria are expressed not with their scientific names, but with the RefSeq accession numbers used by the National Center for Biotechnology Information (NCBI), as shown in Supplementary Table S1. We obtained the protein-domain information for each of the 367 species, as described in reference [15], and then all of the bacteria genome sequences of RefSeq were downloaded from 659 files to obtain the Refseq protein IDs. We determined the protein IDs for 326 species, and for the remaining 41 species, no protein ID was obtained, see Supplementary Table S2. Amino acid sequences of bacteria proteins were used from 398 RefSeq files. We divided these files so that one file contained one amino acid sequence, named as “(protein_id).txt”. The amino acid sequences were searched using the protein ID in the Refseq file for the 326 bacteria species. As shown in Supplementary Table S3, all of the amino acid sequences that correspond to their respective protein IDs were picked up. The following formula derives the percentage of the encoding region of the genome:

Ratio (%) of the coding region on the genome = {(Total length of all amino acid sequences of all proteins) × 3/(length of genome DNA)} × 100

If all (or almost all) of the protein IDs are not picked up through the previous step, the sum of the lengths of the amino acid sequences would be smaller than in reality; therefore, the value would be much smaller than 100%.

Supplementary Table S4 shows the proportions (%) of the coding regions of the respective bacteria that were analyzed. Although NC_005070 showed a proportion of approximately 2.75%, almost all of the species had proportions in the range of 70–90%, and none of the species showed more than 100%. Next, NC_005070 was excluded, and additional analysis was performed for the remaining 325 bacteria species. The domain information was then obtained for each of the 325 species from the respective files that summarize all of the amino acid sequences using Pfam in InterProScan 5.19–58.0 [18].

The results of the analysis were output in a CSV (comma-separated values) file containing 11 columns (15 if options were included) as follows: (1) The protein’s accession ID; (2) the amino acid sequence in the MD5 format; (3) the length of the amino acid sequence; (4) the database used for the analysis; (5) the protein-domain ID in the database; (6) the name of the protein domain; (7) the starting position of the protein domain on the amino acid sequence; (8) the end position of the protein domain on the amino acid sequence; (9) the expected e-value of the protein domain; (10) whether the protein domain is matched (matched = T: true); (11) the analysis data. This research used the protein domain ID and the name of the protein domain. We divided the file into columns and provided the respective species, the types, and the numbers of the domain from Refseqs. This file had 8596 lines, 325 columns, and 8596 protein domains, see Supplementary Table S5.

We made a cluster dendrogram for the pvclust package of the R data-analysis software [19,20,21]. The correlation coefficients were also calculated for all combinations of two species of the 325 species (325C2 = 52,650 patterns). The cluster dendrogram is presented in Supplementary Figure S1. Furthermore, additional CSV files were created, where all of the values were converted into 0 or 1 (1 for larger than 1) and also into a natural logarithm (ln) (after adding 1 to the original value). For each of these two CSV files, see Supplementary Tables S6 and S7, respective cluster dendrograms were created and correlation coefficients were similarly calculated.

### 2.2. Generating a Cluster Dendrogram about Protein Domains Translated from Random 100 bps DNA Fragments

For the 325 genomes acquired from the NCBI RefSeq, 100 bp fragments were randomly cut from the whole genome sequences so that the total length of the fragments would be ten times the length. The sequence of the fragments was translated into amino acid sequences of 6-frame. From these amino acid sequences, we searched for the protein domains using the InterProScan. Next, we created a matrix that showed the sum of the numbers of each domain that was found in the proteins of each species. Then, two files were created. The first had the values 1 or 0 and, where the number of a domain was 1 or more, the value was 1. The other file showed a logarithmic value of “the original number of domain plus 1.” A phylogenetic tree was similarly created to calculate the correlation coefficients for combinations of protein domains using those files.

### 2.3. Generating Phylogenetic Trees from 16S Ribosomal RNA

Bacterial 16S rRNA sequences were using NCBI RefSeq for 325 species. We confirmed the existence of 16S rRNA sequences by searching the corresponding genome. A phylogenetic tree was created based on the DNA sequences of these 16S rRNA sequences for 325 bacteria species using the maximum likelihood method for using Molecular Evolutionary Genetics Analysis 7 (MEGA 7) [22,23]. The phylogenetic tree of 325 species is shown in Supplementary Figure S2.

### 2.4. Generating Phylogenetic Trees for DNA and Amino Acid Sequences of DNA Gyrase Subunit B

We also obtained the gene sequences of 296 species within the 325 species. Supplementary Figure S3 shows the phylogenetic tree that was created for the genome DNA sequences of DNA gyrase subunit B for using MEGA 7. Pairwise distances were also calculated for combinations of two species out of 296 species (_296_C_2_ = 43,660 patterns) [24].

An amino acid sequences phylogenetic tree was also created for DNA gyrase subunit B. RefSeqs divided each of them into files using one protein ID. Among the 296 species, three sequences (NC_ 003028, NC_00527 and NC_013928) were very short. We also found that six sequences (NC_008750, NC_009438, NC_007356, NC_009455, NC_008146 and NC_008705) were exactly the same, and therefore, these six species were excluded from further analyses. A phylogenetic tree was created for the amino acid sequences of DNA gyrase subunit B of those 287 species using MEGA 7, see Supplementary Figure S4.

### 2.5. Comparing the Cluster Dendrograms and Phylogenetic Trees

To compare the cluster dendrograms and the phylogenetic trees, the correlation coefficients for the domain combinations and the pairwise distances for index sequences (DNA sequences for 16S rRNA and DNA and amino acid sequences for DNA gyrase subunit B) were calculated. Those results were dot-plotted using software R for every combination of two among the 322 species.

### 2.6. Analysis Test on the Environmental Data

The 143 environmental metagenome data were downloaded from the DNA Data Bank of Japan (DDBJ) sequence read archive (DDBJ SRA: DRA005425). These data were obtained by periodically collecting seawater from March 2012 to May 2014 in the northeastern coastal area of Japan. After the seawater was filtered using 0.2, 0.8, and 5 μm pore filters, the DNA samples were extracted from the bacteria that was attached to the filter. The 100 bp paired end sequencing was performed with the illumina Hiseq 2000 sequencer (Illumina, San Diego, CA, USA). Information on the collection date, filter size, sampling point and sampling depth is shown in Supplementary Figure S6 and Table S8. Similar to the above method, 100 bp sequence data were translated to amino acid sequences and the protein domain information was searched by Pfam using InterProScan 5.19–58.0. We obtained 500,000 reads, from which sequences of 30 amino acid residues or more were found, and we used these as a test data set. Then, these data were counted for each sample, and the appearance frequency of each motif for each of the samples was matrixed. For this matrix, a heat map analysis using a “gplots” package [25], a cluster analysis using a “pvclast” package [19], and a principal component analysis (PCA) using a “scatterplot3d” package [26] were performed in R software.

## 3. Results

### 3.1. Comparing the Cluster Dendrogram of Domain Combinations and the Phylogenetic Tree of DNA Sequences for 16S Ribosomal RNA

**Figure 1:**
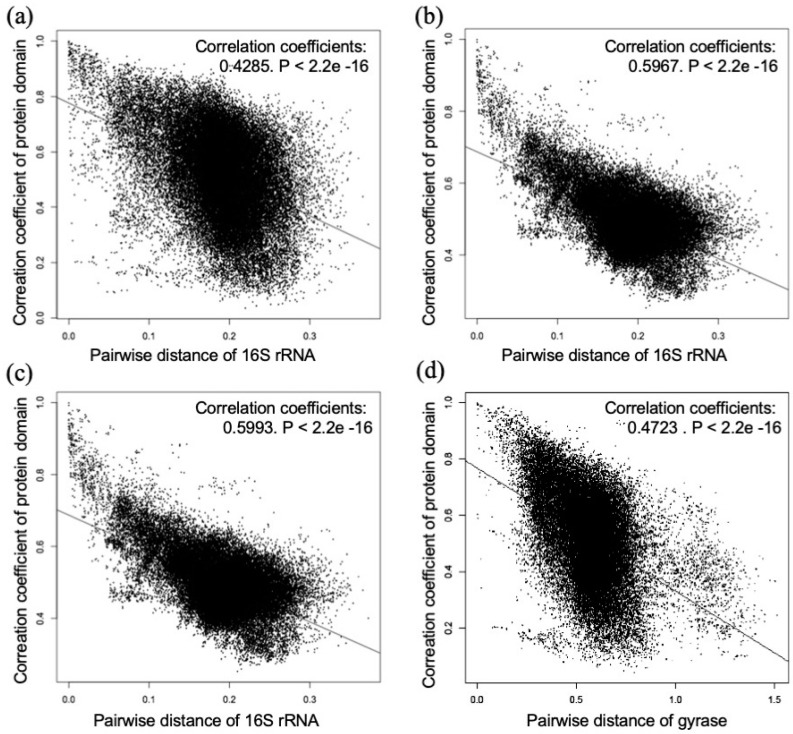
Dot plots for correlation coefficients of domain combinations and pairwise distances of DNA sequences: (**a**) The pairwise distances were calculated based on the 16S rRNA sequence. The correlation coefficient was 0.4285, P < 2.2e−16; (**b**) Domain counts were converted to 0 (absence)/1 (presence), and pairwise distances were calculated based on the 16S rRNA sequence. The correlation coefficient was 0.5967, P < 2.2e−16; (**c**) Domain counts were converted to ln [number of domain + 1], and pairwise distances were calculated based on the 16S rRNA sequence. The correlation coefficient was 0.5993, P < 2.2e−16; and (**d**) The pairwise distances were calculated based on the DNA gyrase subunit B sequence. The correlation coefficient was 0.4723, P < 2.2e−16.

The protein domains were obtained from 325 species. A total of 322 species were used for comparison (except for three that formed clusters that were not close to other species in the phylogenetic tree generated for the DNA sequences of 16S rRNA). The correlation coefficients of the domain combinations and the pairwise distances of the DNA sequences of 16S rRNA were dot-plotted using R for all of the combinations of any two species among the 322 species. The results are presented in Figure 1a. The correlation coefficient was 0.4285, P < 2.2e−16, showing a moderate correlation. Based on this result, it seems that the cluster dendrograms that are based on the domain combinations and the phylogenetic tree that is based on DNA sequences of 16S rRNA are similar to some extent.

### 3.2. Comparing the Cluster Dendrogram for the Existence of Protein Domains, or Converted into Natural Logarithms, with the Phylogenetic Tree Created from DNA Sequences of 16S Ribosomal RNA

The correlation coefficients were obtained for each of the two data sets—one in which all of the values were converted to 0 (absence) or 1 (presence), see Supplementary Table S6 and another in which all of the values were converted into a ln (original value + 1), see Supplementary Table S7—and the pairwise distances of the DNA sequences of 16S rRNA were compared for every combination of two species among the 230 species. Then, they were analyzed, as was previously described. Figure 1b shows a dot plot that uses correlation coefficients of the file that represents the number of the domain as 0 (absence) or 1 (presence), and Figure 1c shows the same result using the correlation coefficients of the file with a ln (number of domain + 1) for 16S rRNA. The correlation coefficients for Figure 1b,c are 0.5967 and 0.5993, respectively, and both indicate a strong correlation. The P-value for Figure 1b,c is <2.2e−16.

### 3.3. Comparison of the Cluster Dendrogram Based on Protein Domains Translated from Random 100 bp DNA Fragments and that Created from 16S Ribosomal RNA

Similarly, we made comparisons based on the information for 16S rRNA. Supplementary Figure S5a shows the correlation between the domain combinations and the pairwise distance for 16S rRNA with fragments of 100 bp. Using DNA fragments of 100 bp, the correlation coefficient was 0.4425, which shows a moderate correlation, and the P-value was <2.2e−16.

Supplementary Figure S5b,c presents the results when the number of domains was converted to either 1 (existence) or 0 (absence), see Supplementary Figure S5b, or converted to a natural logarithmic value of the number + 1, see Supplementary Figure S5c. When comparing a phylogenetic tree that was created by extracting the protein domains from fragments of 100 bp, in which the numbers of the domains were converted into either 1 (existence) or 0 (absence), to that created from 16S rRNA, see Supplementary Figure S5b, the correlation coefficient was 0.4775 and the P-value was < 2.2e−16. Similarly, with the numbers of the domains converted to a natural logarithmic value of the original number + 1, see Supplementary Figure S5c, the correlation coefficient was 0.5921 and the P-value was <2.2e−16.

### 3.4. Comparison of the Correlation Coefficients of the Domain Combinations and Pairwise Distances of 16S Ribosomal RNA and DNA Gyrase Subunit B

For 296 species from which DNA sequences of DNA gyrase subunit B were obtained, the correlation coefficients for the domain combinations and pairwise distances of DNA sequences of DNA gyrase subunit B were similarly dot-plotted using R. Furthermore, for 284 species for which the amino acid sequences of the DNA gyrase subunit B were obtained (the three species that formed a cluster apart from the other 284 species in the created tree were excluded), the correlation coefficients for the domain combinations and pairwise distances of the amino acid sequences of the DNA gyrase subunit B were similarly dot-plotted using R.

The results are shown in Figure 1d for the DNA sequences and in Supplementary Figure S5d for the amino acid sequences. The correlation coefficients were 0.4723 and 0.4656, respectively, which indicates moderate correlation. The P-value for Figure 1d and Supplementary Figure S5d is <2.2e−16.

### 3.5. Cluster Analysis and Principal Component Analysis of the Environmental Data

**Figure 2:**
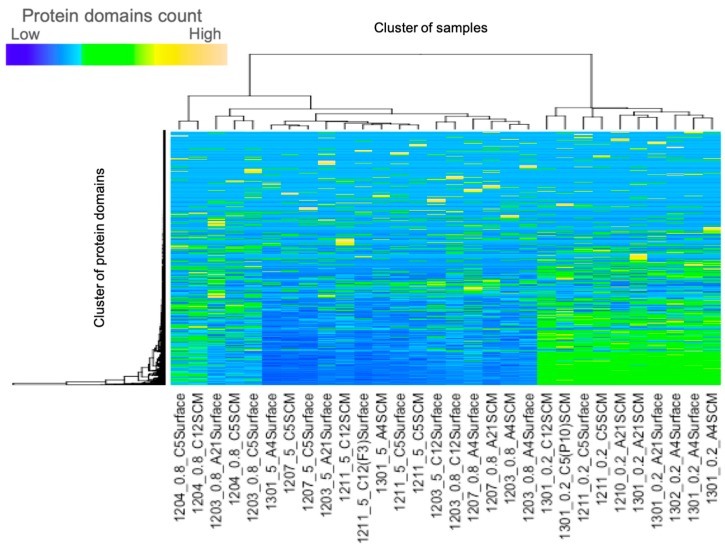
Heatmap analysis of the protein domains using 30 samples of the environmental metagenomic data. It is divided into two large clusters: Clusters of 5 μm and 0.8 μm samples on the left cluster, while the right cluster contains 0.2 μm samples. See Supplementary Figure S7 for an analysis of the results using all of the data sets.

In each of the 500,000 reads from the metagenome data from the northeastern coastal area of Japan, the average number of reads for which the protein domains were identified by Pfam were 50,089 (10.02%), 19,566 (3.91%) and 8093 (1.62%) for the 0.2, 0.8 and 5 μm filters, respectively, and this suggests that more motif’s data were identified from samples using a smaller filter size. A heat map analysis that calculates the frequency of occurrence of each domain indicated that most of the samples were clustered by the sizes of the filters, see Figure 2, Supplementary Figure S7.

**Figure 3:**
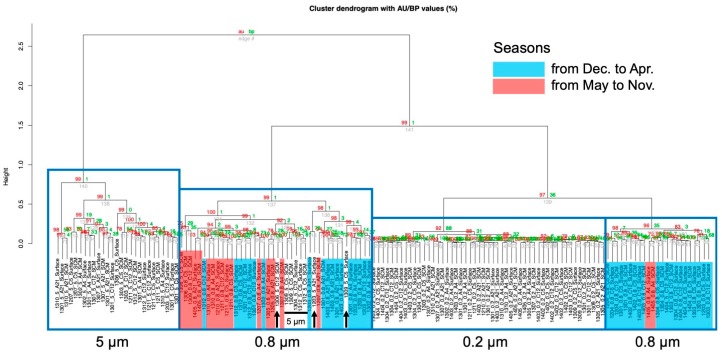
Cluster analysis based on the protein domains using environmental metagenomic data. The distance between the samples was calculated by correlating the distance and they were clustered using the “ward.D2” method. It is divided into four clusters. The black bars and arrows indicate 5 μm filter samples in a 0.8 μm filter sample. See Supplementary Figure S8 for the high-resolution version.

Subsequently, a cluster analysis was carried out in detail using the pvclust package. As a result, the samples were divided into four clusters, which were corresponded to the filters’ sizes, one to 0.2 μm, another to 5 μm and the other two clusters to two 0.8 μm, see Figure 3 and Supplementary Figure S8, except for some samples. These results suggested that the composition of the protein motifs of the organisms were grouped according to size. Within the two clusters of 0.8 μm, a seasonal factor appeared to be a moderate factor in the creation of the form groups, and this is apparent because most of the samples in one cluster consisted of low-temperature seasons.

**Figure 4:**
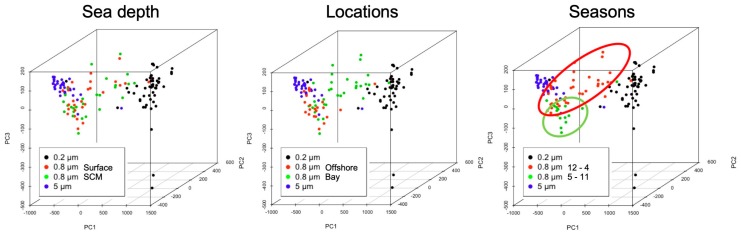
A principal component analysis was carried out on the protein domains by the environmental data. The data of the 0.8 μm filter samples were examined under three conditions: Sea depth, namely surface (1 m) vs. SCM (10–20 m); locations, namely the bay vs. the offshore area; the season, namely from December to April vs. from May to November. The red and green circles show samples from December to April and from May to November, respectively.

Finally, a principal component analysis (PCA) was performed in order to determine the factor that caused the 0.8 μm samples to split into two clusters. Sea depth (surface (1 m) vs. surface chlorophyll maxima; SCM (10–20 m)), see Supplementary Table S8, locations (bay vs. offshore area), see Supplementary Figure S7, and the season (from December to April vs. from May to November) were investigated. As a result, the 0.8 μm samples were most clearly divided into two groups when compared with the seasons, see Figure 4.

## 4. Discussion

The correlation analysis revealed that the cluster dendrogram that was generated based on the combination of the protein domains is somewhat similar to the phylogenetic tree for the DNA or amino acid sequence of 16S rRNA and DNA gyrase subunit B, see Figure 1. This result suggests that the combinations of the protein domains are similar among phylogenetically close species. Moreover, the correlation coefficients were higher in the two dot plots, see Figure 1b,c, which suggests that, by eliminating or reducing the influence of the domains that a given bacterium has in large amounts, the cluster dendrogram will become similar to the phylogenetic tree that is generated based on the DNA sequences of 16S rRNA. Therefore, when analyzing the protein domain, it is apparent that the protein domains that occurred in a large amount in a given bacterium should be excluded for an effective analysis. In addition, the present study revealed that the compositions of the protein domains are similar across evolutionally close bacteria. Since the number of various protein domains differs from bacterium to bacterium in the above analyses, it should be converted to, for example, its natural logarithm. Furthermore, the results were similar when we analyzed a protein domain from randomly selected DNA fragments, see Supplementary Figure S5. Therefore, this method also appears to be effective for data with a short DNA sequence of about 100 bp that is randomly obtained from a next-generation sequencer.

In order to verify the effectiveness of the protein motif-based method, we used actual environmental data from the Tohoku coastal area of Japan. The filter samples with larger pore sizes had fewer protein domains, as found by the Pfam database search. Other studies explain that this result is due to the large number of DNA sequences that are derived from the phytoplankton in the filter with a large pore size, and therefore, the sequence of the metagenome might contain a large amount of non-coding regions intron and intergenic sequences [27,28].

A PCA analysis was performed to determine a factor of 0.8 μm pore filter samples, which were divided into two clusters by cluster analysis. When the samples were classified by seasons, the samples were plotted more accurately, see Figure 4. It has been reported that the microbial flora changes seasonally in this area [29,30]. Therefore, it appears that the factor that can be used to cluster the 0.8 μm samples into two is the microbial phase shift that accompanies a change of the seasons, and this suggests that the analytical method using a combination of protein domains would allow us to consider what kind of environment the microorganisms originate. This technique would be helpful for picking undiscovered information and/or providing additional information from metagenomic data.

## Supplementary Materials

The following are available online at https://www.mdpi.com/2227-7382/7/2/19/s1: Figure S1: Cluster dendrogram based on combinations of protein domains for 326 bacteria species; Figure S2: A phylogenetic tree that is based on the 16S rRNA sequences of 325 bacteria species; Figure S3: A phylogenetic tree that was created using the genome DNA sequences of DNA gyrase subunit B for 296 species; Figure S4: A phylogenetic tree that is based on the amino-acid sequences of DNA gyrase subunit B for 296 species; Figure S5: A dot plot for the correlation coefficients of domain combinations that are converted from 100 bp DNA fragments and pairwise distances; Figure S6: Sampling location of environmental DNA; Figure S7: A heatmap analysis about the protein domains that uses a full set of environmental metagenomic data; Figure S8: Cluster analysis based on the protein domains using environmental metagenomic data; Table S1: A list of names and NCBI accession numbers of bacteria that are used in the present analysis; Table S2: A list of success or failure rates when obtaining protein_ids; Table S3: The percentage of protein_ids that are picked-up for the respective species; Table S4: The proportions of the coding regions of the bacteria that were analyzed; Table S5: The number of domains that contained in a certain bacterium; Table S6: The number of domains that contained a certain bacterium (all values converted to 0 (absent) or 1 (if larger than 1); Table S7: The number of domains that contained a certain bacterium (all values calculated to ln [original value +1]); and Table S8: The biological information about the metagenomic sequence data.
