# Supplementary material for: A Preliminary Metagenome Analysis Based on a Combination of Protein Domains
Source: Proteomes. 2019 Apr 29;7(2):19. doi: 10.3390/proteomes7020019 (PMC6630717; doi:10.3390/proteomes7020019)
Supplement: Supplementary file 1 [file proteomes-07-00019-s001.zip › supplementary/Figure S5.pptx]

## Slide 1
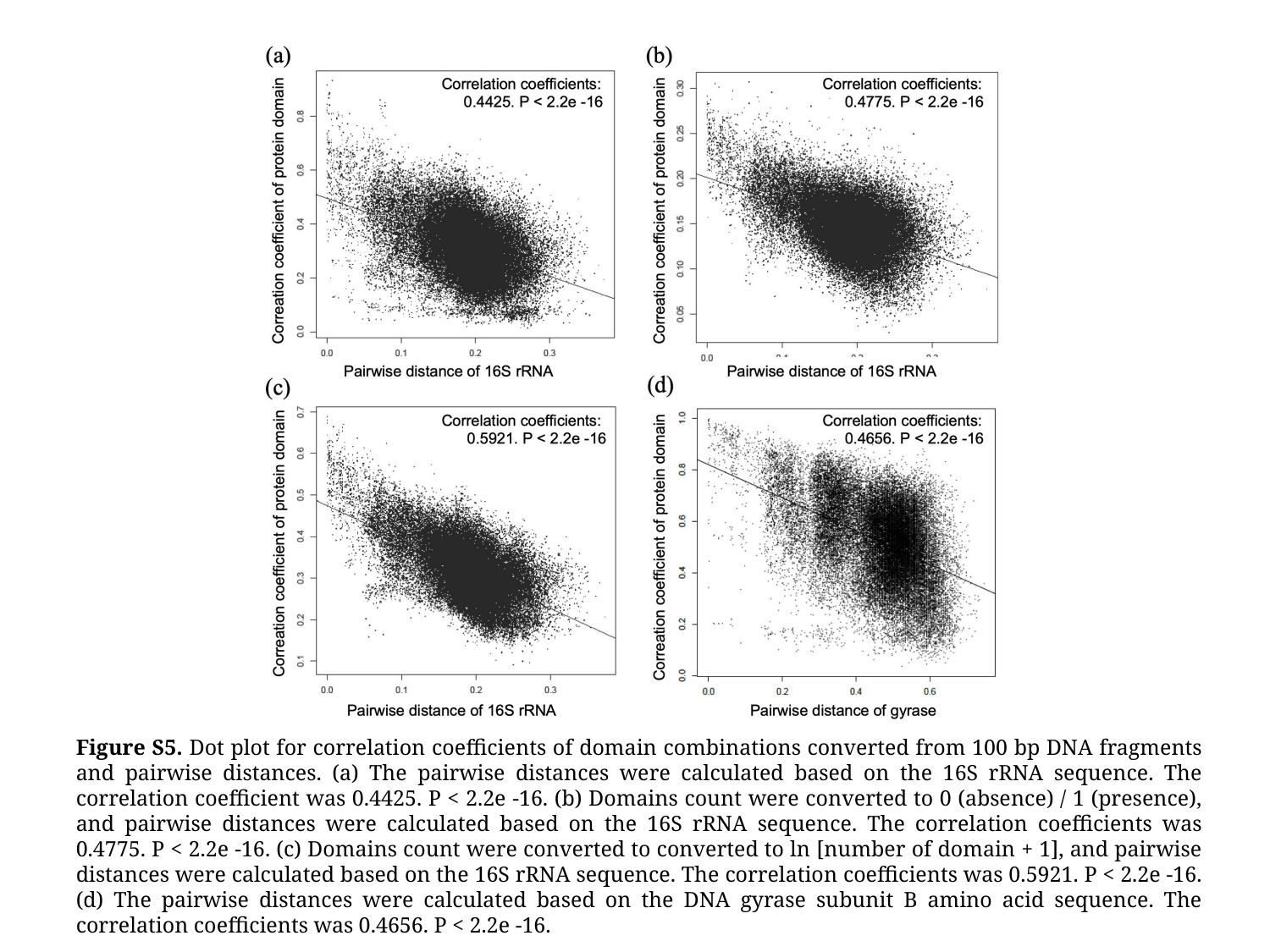

Figure S5. Dot plot for correlation coefficients of domain combinations converted from 100 bp DNA fragments and pairwise distances. (a) The pairwise distances were calculated based on the 16S rRNA sequence. The correlation coefficient was 0.4425. P < 2.2e -16. (b) Domains count were converted to 0 (absence) / 1 (presence), and pairwise distances were calculated based on the 16S rRNA sequence. The correlation coefficients was 0.4775. P < 2.2e -16. (c) Domains count were converted to converted to ln [number of domain + 1], and pairwise distances were calculated based on the 16S rRNA sequence. The correlation coefficients was 0.5921. P < 2.2e -16. (d) The pairwise distances were calculated based on the DNA gyrase subunit B amino acid sequence. The correlation coefficients was 0.4656. P < 2.2e -16.
