# Supplementary material for: A Preliminary Metagenome Analysis Based on a Combination of Protein Domains
Source: Proteomes. 2019 Apr 29;7(2):19. doi: 10.3390/proteomes7020019 (PMC6630717; doi:10.3390/proteomes7020019)
Supplement: Supplementary file 1 [file proteomes-07-00019-s001.zip › supplementary/Figure S3.pptx]

## Slide 1
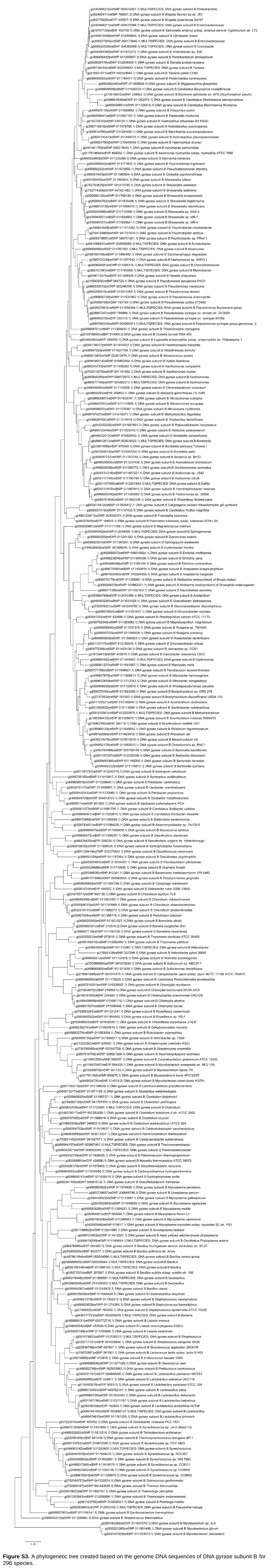

Figure S3. A phylogenetic tree created based on the genome DNA sequences of DNA gyrase subunit B for 296 species.
