# Supplementary material for: A Preliminary Metagenome Analysis Based on a Combination of Protein Domains
Source: Proteomes. 2019 Apr 29;7(2):19. doi: 10.3390/proteomes7020019 (PMC6630717; doi:10.3390/proteomes7020019)
Supplement: Supplementary file 1 [file proteomes-07-00019-s001.zip › supplementary/Table S2.pdf]

**Table S2**. List of success or failure in obtaining protein_ids
T: true (protein_id obtained), F: failed (not obtained)

| Bacteria name | NCBI accession No. | T/F |
|---|---|---|
| Fervidobacterium nodosum Rt17-B1 | NC_009718 | T |
| Petrotoga mobilis SJ95 | NC_010003 | T |
| Thermosipho melanesiensis BI429 | NC_009616 | T |
| Thermotoga lettingae TMO | NC_009828 | T |
| Thermotoga maritima MSB8 | NC_000853 | T |
| Thermotoga petrophila RKU-1 | NC_009486 | T |
| Candidus 'Acertothermus autotrophicum | AP011800-03 | F |
| Deinococcus geothermalis DSM 11300 | NC_008025 | T |
| Deinococcus radiodurans R1 | NC_001263-64 | F |
| Thermus thermophilus HB27 | NC_005835 | T |
| Borrelia afzelii PKo | NC_008277 | T |
| Borrelia burgdorferi B31 | NC_001318 | T |
| Borrelia garinii PBi | NC_006156 | T |
| Leptospira borgpetersenii serovar Hardjo-bovis JB197 | NC_008510-11 | F |
| Treponema denticola ATCC 35405 | NC_002967 | T |
| Treponema pallidumsub sp. pallidum str. Nichols | NC_000919 | T |
| Leptospira interrogans serovar Lai str. 56601 | NC_004342-43 | F |
| Chlorobium chlorochromatii CaD3 | NC_007514 | T |
| Chlorobium phaeobacteroides DSM 266 | NC_008639 | T |
| Chlorobium tepidum TLS | NC_002932 | T |
| Pelodictyon luteolum DSM 273 | NC_007512 | T |
| Prosthecochloris vibrioformis DSM 265 | NC_009337 | T |
| Cytophaga hutchinsonii ATCC 33406 | NC_008255 | T |
| Flavobacterium johnsoniae UW101 | NC_009441 | T |
| Gramella forsetii KT0803 | NC_008571 | T |
| Porphyromonas gingivalis W83 | NC_002950 | T |

Table S2. *(Continued)*

| Bacteria name | NCBI accession No. | T/F |
|---|---|---|
| Salinibacter ruber DSM 13855 | NC_007677 | T |
| Bacteroides fragilis YCH46 | NC_006347 | T |
| Bacteroides thetaiotaomicron VPI-5482 | NC_004663 | T |
| Rhodopirellula baltica SH 1 | NC_005027 | T |
| Candidatus Protochlamydia amoebophila UWE25 | NC_005861 | T |
| Chlamydia muridarum Nigg | NC_002620 | T |
| Chlamydia trachomatis D/UW-3/CX | NC_000117 | T |
| Chlamydophila abortus S26/3 | NC_004552 | T |
| Chlamydophila caviae GPIC | NC_003361 | T |
| Chlamydophila pneumoniae CWL029 | NC_000922 | T |
| Aquifex aeolicus VF5 | NC_000918 | T |
| Arcobacter butzleri RM4018 | NC_009850 | T |
| Campylobacter fetus subsp. fetus 82-40 | NC_008599 | T |
| Campylobacter jejuni subsp. jejuni NCTC 11168 | NC_002163 | T |
| Helicobacter hepaticus ATCC 51449 | NC_004917 | T |
| Helicobacter pylori 26695 | NC_000915 | T |
| Sulfurimonas denitrificans DSM 1251 | NC_007575 | T |
| Sulfurovum sp. NBC37-1 | NC_009663 | T |
| Wolinella succinogenes DSM 1740 | NC_005090 | T |
| Nitratiruptor sp. SB155-2 | NC_009662 | T |
| Anaeromyxobacter sp. Fw109-5 | NC_009675 | T |
| Bdellovibrio bacteriovorus HD100 | NC_005363 | T |
| Desulfococcus oleovorans Hxd3 | NC_009943 | T |
| Desulfotalea psychrophila LSv54 | NC_006138 | T |
| Desulfovibrio desulfuricans G20 | NC_007519 | T |
| Desulfovibrio vulgaris subsp. vulgaris str. Hildenborough | NC_002937 | T |

**Table S2.** *(Continued)*

| Bacteria name | NCBI accession No. | T/F |
|---|---|---|
| Geobacter metallireducens GS-15 | NC_007517 | T |
| Geobacter sulfurreducens PCA | NC_002939 | T |
| Geobacter uraniireducens Rf4 | NC_009483 | T |
| Pelobacter carbinolicus DSM 2380 | NC_007498 | T |
| Pelobacter propionicus DSM 2379 | NC_008609 | T |
| Sorangium cellulosum 'So ce 56' | NC_010162 | T |
| Syntrophobacter fumaroxidans MPOB | NC_008554 | T |
| Syntrophus aciditrophicus SB | NC_007759 | T |
| Myxococcus xanthus DK 1622 | NC_008095 | T |
| Magnetococcus sp. MC-1 | NC_008576 | T |
| Bradyrhizobium japonicum USDA110 | NC_004463 | T |
| Bradyrhizobium sp. ORS278 | NC_009445 | T |
| Brucella abortus biovar 1 str. 9-941 | NC_006932-33 | F |
| Brucella canis ATCC 23365 | NC_010103-104 | F |
| Brucella melitensis 16M | NC_003317-18 | F |
| Brucella ovis ATCC 25840 | NC_009504-05 | F |
| Brucella suis 1330 | NC_004310-11 | F |
| Candidatus Pelagibacter ubique HTCC1062 | NC_007205 | T |
| Caulobacter crescentus CB15 | NC_002696 | T |
| Dinoroseobacter shibae DFL 12 | NC_009952 | T |
| Erythrobacter litoralis HTCC2594 | NC_007722 | T |
| Gluconacetobacter diazotrophicus PAl 5 | NC_010125 | T |
| Gluconobacter oxydans 621H | NC_006677 | T |
| Granulobacter bethesdensis CGDNIH1 | NC_008343 | T |
| Hyphomonas neptunium ATCC 15444 | NC_008358 | T |
| Jannaschia sp. CCS1 | NC_007802 | T |
| Ochrobactrum anthropi ATCC 49188 | NC_009667-68 | F |

Table S2. *(Continued)*

| Bacteria name | NCBI accession No. | T/F |
|---|---|---|
| Parvibaculum lavamentivorans DS-1 | NC_009719 | T |
| Rhizobium etli CFN 42 | NC_007761 | T |
| Rhizobium leguminosarum bv.viciae 3841 | NC_008380 | T |
| Rhodobacter sphaeroides 2.4.1 | NC_007493-94 | F |
| Rhodopseudomonas palustris CGA009 | NC_005296 | T |
| Rhodospirillum rubrum ATCC 11170 | NC_007643 | T |
| Roseobacter denitrificans OCh 114 | NC_008209 | T |
| Rubrobacter xylanophilus DSM 9941 | NC_008148 | T |
| Silicibacter pomeroyi DSS-3 | NC_003911 | T |
| Silicibacter sp. TM1040 | NC_008044 | T |
| Sinorhizobium medicae WSM419 | NC_009636 | T |
| Sinorhizobium meliloti 1021 | NC_003047 | T |
| Sphingomonas wittichii RW1 | NC_009511 | T |
| Sphingopyxis alaskensis RB2256 | NC_008048 | T |
| Wolbachia endosymbiont of Drosophila melanogaster | NC_002978 | T |
| Wolbachia endosymbiont strain TRS of Brugia malayi | NC_006833 | T |
| Xanthobacter autotrophicus Py2 | NC_009720 | T |
| Zymomonas mobilis subsp. mobilis ZM4 | NC_006526 | T |
| Magnetospirillum magneticum AMB-1 | NC_007626 | T |
| Maricaulis maris MCS10 | NC_008347 | T |
| Mesorhizobium loti MAFF303099 | NC_002678 | T |
| Mesorhizobium sp. BNC1 | NC_008254 | T |
| Methylobacterium extorquens PA1 | NC_010172 | T |
| Neorickettsia sennetsu str. Miyayama | NC_007798 | T |
| Nitrobacter hamburgensis X14 | NC_007964 | T |
| Nitrobacter winogradskyi Nb-255 | NC_007406 | T |
| Acidiphilium cryptum JF-5 | NC_009484 | T |

Table S2. *(Continued)*

| Bacteria name | NCBI accession No. | T/F |
|---|---|---|
| Agrobacterium tumefaciens str. C58 | NC_003062-63 | F |
| Anaplasma marginale str. St. Maries | NC_004842 | T |
| Anaplasma phagocytophilum HZ | NC_007797 | T |
| Azorhizobium caulinodans ORS 571 | NC_009937 | T |
| Bartonella bacilliformis KC583 | NC_008783 | T |
| Bartonella henselae str. Houston-1 | NC_005956 | T |
| Bartonella quintana str. Toulouse | NC_005955 | T |
| Bartonella tribocorum CIP 105476 | NC_010161 | T |
| Acidovorax avenae subsp. citrulli AAC00-1 | NC_008752 | T |
| Acidovorax sp. JS42 | NC_008782 | T |
| Azoarcus sp. BH72 | NC_008702 | T |
| Azoarcus sp. EbN1 | NC_006513 | T |
| Bordetella bronchiseptica RB50 | NC_002927 | T |
| Bordetella parapertussis 12822 | NC_002928 | T |
| Bordetella pertussis Tohama I | NC_002929 | T |
| Bordetella petrii DSM 12804 | NC_010170 | T |
| Burkholderia ambifaria AMMD | NC_010551-52,57 | F |
| Burkholderia cenocepacia AU 1054 | NC_008060-62 | F |
| Burkholderia multivorans ATCC 17616 | NC_010086-87. 84 | F |
| Burkholderia pseudomallei K96243 | NC_006350-51 | F |
| Burkholderia sp. 383 | NC_007509-11 | F |
| Burkholderia thailandensis E264 | NC_007650-51 | F |
| Burkholderia vietnamiensis G4 | NC_009254-56 | F |
| Burkholderia xenovorans LB400 | NC_007951-53 | F |
| Chromobacterium violaceum ATCC 12472 | NC_005085 | T |
| Dechloromonas aromatica RCB | NC_007298 | T |

Table S2. *(Continued)*

| Bacteria name | NCBI accession No. | T/F |
|---|---|---|
| Delftia acidovorans SPH-1 | NC_010002 | T |
| Nitrosomonas europaea ATCC 19718 | NC_004757 | T |
| Nitrosomonas eutropha C91 | NC_008344 | T |
| Nitrosospira multiformis ATCC 25196 | NC_007614 | T |
| Polaromonas sp. JS666 | NC_007948 | T |
| Polynucleobacter sp. QLW-P1DMWA-1 | NC_009379 | T |
| Ralstonia solanacearum GMI1000 | NC_003295 | T |
| Rhodoferax ferrireducens T118 | NC_007908 | T |
| Thiobacillus denitrificans ATCC 25259 | NC_007404 | T |
| Verminephrobacter eiseniae EF01-2 | NC_008786 | T |
| Methylobacillus flagellatus KT | NC_007947 | T |
| Neisseria gonorrhoeae FA 1090 | NC_002946 | T |
| Neisseria meningitidis MC58 | NC_002946 | T |
| Acinetobacter sp. ADP1 | NC_005966 | T |
| Actinobacillus pleuropneumoniae L20 | NC_009053 | T |
| Actinobacillus succinogenes 130Z | NC_009655 | T |
| Aeromonas hydrophila subsp. hydrophila ATCC 7966 | NC_008570 | T |
| Aeromonas salmonicida subsp. salmonicida A449 | NC_009348 | T |
| Alcanivorax borkumensis SK2 | NC_008260 | T |
| Alkalilimnicola ehrlichei MLHE-1 | NC_008340 | T |
| Baumannia cicadellinicola str. Hc (Homalodisca coagulata) | NC_007984 | T |
| Buchnera aphidicola str. APS (Acyrthosiphon pisum) | NC_002528 | T |
| Candidatus Blochmannia floridanus | NC_005061 | T |
| Candidatus Blochmannia pennsylvanicus str. BPEN | NC_007292 | T |
| Candidatus Ruthia magnifica str. Cm (Calyptogena magnifica) | NC_008610 | T |
| Candidatus Vesicomyosocius okutanii HA | NC_009465 | T |

Table S2. *(Continued)*

| Bacteria name | NCBI accession No. | T/F |
|---|---|---|
| Chromohalobacter salexigens DSM 3043 | NC_007963 | T |
| Citrobacter koseri ATCC BAA-895 | NC_009792 | T |
| Colwellia psychrerythraea 34H | NC_003910 | T |
| Coxiella burnetii RSA 493 | NC_002971 | T |
| Ehrlichia canis str. Jake | NC_007354 | T |
| Ehrlichia chaffeensis str. Arkansas | NC_007799 | T |
| Ehrlichia ruminantium str. Welgevonden | NC_005295 | T |
| Enterobacter sakazakii ATCC BAA-894 | NC_009778 | T |
| Enterobacter sp. 638 | NC_009436 | T |
| Erwinia carotovora subsp. atroseptica SCRI1043 | NC_004547 | T |
| Escherichia coli K-12 MG1655 | NC_000913 | T |
| Francisella tularensis subsp. novicida U112 | NC_008601 | T |
| Francisella tularensis subsp. tularensis SCHU S4 | NC_006570 | T |
| Haemophilus ducreyi 35000HP | NC_002940 | T |
| Haemophilus influenzae Rd KW20 | NC_000907 | T |
| Haemophilus somnus 129PT | NC_008309 | T |
| Hahella chejuensis KCTC 2396 | NC_007645 | T |
| Halorhodospira halophila SL1 | NC_008789 | T |
| Idiomarina loihiensis L2TR | NC_006512 | T |
| Klebsiella pneumoniae subsp. pneumoniae MGH 78578 | NC_009648 | T |
| Legionella pneumophila subsp. pneumophila str. Philadelphia 1 | NC_002942 | T |
| Pasteurella multocida subsp. multocidastr. Pm70 | NC_002663 | T |
| Photobacterium profundum SS9 | NC_006370-71 | F |
| Photorhabdus luminescens subsp. laumondii TTO1 | NC_005126 | T |
| Pseudoalteromonas atlantica T6c | NC_008228 | T |
| Pseudoalteromonas haloplanktis TAC125 | NC_007481-82 | F |

Table S2. *(Continued)*

| Bacteria name | NCBI accession No. | T/F |
|---|---|---|
| Pseudomonas aeruginosa PAO1 | NC_002516 | T |
| Pseudomonas entomophila L48 | NC_008027 | T |
| Pseudomonas fluorescens Pf-5 | NC_004129 | T |
| Pseudomonas mendocina ymp | NC_009439 | T |
| Pseudomonas putida KT2440 | NC_002947 | T |
| Pseudomonas stutzeri A1501 | NC_009434 | T |
| Pseudomonas syringae pv. phaseolicola 1448A | NC_005773 | T |
| Pseudomonas syringae pv. syringae B728a | NC_007005 | T |
| Pseudomonas syringae pv. tomato str. DC3000 | NC_004578 | T |
| Psychrobacter arcticus 273-4 | NC_007204 | T |
| Psychrobacter cryohalolentis K5 | NC_007969 | T |
| Psychrobacter sp. PRwf-1 | NC_009524 | T |
| Psychromonas ingrahamii 37 | NC_008709 | T |
| Saccharophagus degradans 2-40 | NC_007912 | T |
| Salmonella typhimurium LT2 | NC_003197 | T |
| Serratia proteamaculans 568 | NC_009832 | T |
| Shewanella amazonensis SB2B | NC_008700 | T |
| Shewanella denitrificans OS217 | NC_007954 | T |
| Shewanella frigidimarina NCIMB 400 | NC_008345 | T |
| Shewanella loihica PV-4 | NC_009092 | T |
| Shewanella oneidensis MR-1 | NC_004347 | T |
| Shewanella pealeana ATCC 700345 | NC_009901 | T |
| Shewanella putrefaciens CN-32 | NC_009438 | T |
| Shewanella sediminis HAW-EB3 | NC_009831 | T |
| Shewanella sp. ANA-3 | NC_008577 | T |
| Shewanella sp. MR-4 | NC_008321 | T |
| Shewanella sp. MR-7 | NC_008322 | T |

Table S2. *(Continued)*

| Bacteria name | NCBI accession No. | T/F |
|---|---|---|
| Shewanella sp. W3-18-1 | NC_008750 | T |
| Shigella boydii Sb227 | NC_007613 | T |
| Shigella dysenteriae Sd197 | NC_007606 | T |
| Shigella flexneri 2a str. 301 | NC_004337 | T |
| Shigella sonnei Ss046 | NC_007384 | T |
| Thiomicrospira crunogena XCL-2 | NC_007520 | T |
| Vibrio cholerae O1 biovar eltor str. N16961 | NC_002505-06 | F |
| Vibrio fischeri ES114 | NC_006840-41 | F |
| Vibrio harveyi ATCC BAA-1116 | NC_009783-84 | F |
| Vibrio vulnificus CMCP6 | NC_004459-60 | F |
| Wigglesworthia glossinidia endosymbiont of Glossina brevipalpis | NC_004344 | T |
| Xanthomonas axonopodis pv. citristr. 306 | NC_003919 | T |
| Xanthomonas campestris pv. campestris str. ATCC 33913 | NC_007086 | T |
| Xanthomonas campestris pv. vesicatoria str. 85-10 | NC_007508 | T |
| Xanthomonas oryzae pv. oryzae KACC10331 | NC_006834 | T |
| Xylella fastidiosa 9a5c | NC_002488 | T |
| Yersinia enterocolitica subsp. enterocolitica 8081 | NC_008800,NC_008791 | F |
| Yersinia pestis CO92 | NC_003143 | T |
| Yersinia pseudotuberculosis IP 32953 | NC_006155 | T |
| Mannheimia succiniciproducens MBEL55E | NC_006300 | T |
| Marinobacter aquaeolei VT8 | NC_008740 | T |
| Marinomonas sp. MWYL1 | NC_009654 | T |
| Nitrosococcus oceani ATCC 19707 | NC_007484 | T |
| Solibacter usitatus Ellin6076 | NC_008536 | T |
| Acidobacteria bacterium Ellin345 | NC_008009 | T |

**Table S2.** *(Continued)*

| Bacteria name | NCBI accession No. | T/F |
|---|---|---|
| Onion yellows phytoplasma OY-M | NC_005303 | T |
| Ureaplasma parvumserovar 3 str. ATCC 700970 | NC_002162 | T |
| Acholeplasma laidlawii PG-8A | NC_010163 | T |
| Aster yellows witches'-broom phytoplasma AYWB | NC_007716 | T |
| Mesoplasma florum L1 | NC_006055 | T |
| Mycoplasma agalactiae PG2 | NC_009497 | T |
| Mycoplasma capricolum subsp. capricolum ATCC 27343 | NC_007633 | T |
| Mycoplasma gallisepticum R | NC_004829 | T |
| Mycoplasma hyopneumoniae 232 | NC_006360 | T |
| Mycoplasma mobile 163K | NC_006908 | T |
| Mycoplasma mycoides subsp. mycoides SC str. PG1 | NC_005364 | T |
| Mycoplasma penetrans HF-2 | NC_004432 | T |
| Caldicellulosiruptor saccharolyticus DSM 8903 | NC_009437 | T |
| Carboxydothermus hydrogenoformans Z-2901 | NC_007503 | T |
| Clostridium acetobutylicum ATCC 824 | NC_003030 | T |
| Clostridium beijerinckii NCIMB 8052 | NC_009617 | T |
| Clostridium botulinum A str. ATCC 3502 | NC_009495 | T |
| Clostridium kluyveri DSM 555 | NC_009706 | T |
| Clostridium novyi NT | NC_008593 | T |
| Clostridium perfringens str. 13 | NC_003366 | T |
| Clostridium phytofermentans ISDg | NC_010001 | T |
| Clostridium thermocellum ATCC 27405 | NC_009012 | T |
| Desulfitobacterium hafniense Y51 | NC_007907 | T |
| Desulfotomaculum reducens MI-1 | NC_009253 | T |
| Enterococcus faecalis V583 | NC_004668 | T |
| Geobacillus kaustophilus HTA426 | NC_006510 | T |
| Geobacillus thermodenitrificans NG80-2 | NC_009328 | T |

Table S2. *(Continued)*

| Bacteria name | NCBI accession No. | T/F |
|---|---|---|
| Lactobacillus acidophilus NCFM | NC_006814 | T |
| Lactobacillus casei ATCC 334 | NC_008526 | T |
| Lactobacillus delbrueckii subsp. bulgaricus ATCC 11842 | NC_008054 | T |
| Lactobacillus gasseri ATCC 33323 | NC_008530 | T |
| Lactobacillus helveticus DPC 4571 | NC_010080 | T |
| Lactobacillus johnsonii NCC 533 | NC_005362 | T |
| Lactobacillus plantarum WCFS1 | NC_004567 | T |
| Lactobacillus sakei subsp. Sakei 23K | NC_007576 | T |
| Lactobacillus salivarius UCC118 | NC_007929 | T |
| Lactococcus lactis subsp. lactis IL1403 | NC_002662 | T |
| Oceanobacillus iheyensis HTE831 | NC_004193 | T |
| Oenococcus oeni PSU-1 | NC_008528 | T |
| Pediococcus pentosaceus ATCC 25745 | NC_008525 | T |
| Pelotomaculum thermopropionicum SI | NC_009454 | T |
| Staphylococcus aureus subsp. aureus N315 | NC_002745 | T |
| Staphylococcus epidermidis ATCC 12228 | NC_004461 | T |
| Staphylococcus haemolyticus JCSC1435 | NC_007168 | T |
| Staphylococcus saprophyticus subsp. saprophyticus ATCC 15305 | NC_007350 | T |
| Streptococcus agalactiae 2603V/R | NC_004116 | T |
| Streptococcus gordonii str. Challis substr. CH1 | NC_009785 | T |
| Streptococcus mutans UA159 | NC_013928 | T |
| Streptococcus pneumoniae TIGR4 | NC_003028 | T |
| Streptococcus pyogenes M1 GAS | NC_002737 | T |
| Streptococcus sanguinis SK36 | NC_009009 | T |
| Streptococcus thermophilus CNRZ1066 | NC_006449 | T |
| Symbiobacterium thermophilum IAM 14863 | NC_006177 | T |

Table S2. *(Continued)*

| Bacteria name | NCBI accession No. | T/F |
|---|---|---|
| Syntrophomonas wolfei subsp. wolfeistr. Goettingen | NC_008346 | T |
| Thermoanaerobacter ethanolicus ATCC 33223 | NC_010321 | T |
| Thermoanaerobacter ethanolicus X514 | NC_010320 | T |
| Thermoanaerobacter tengcongensis MB4 | NC_003869 | T |
| Alkaliphilus metalliredigens QYMF | NC_009633 | T |
| Bacillus amyloliquefaciens FZB42 | NC_009725 | T |
| Bacillus anthracis str. Ames | NC_003997 | T |
| Bacillus clausii KSM-K16 | NC_006582 | T |
| Bacillus licheniformis ATCC 14580 | NC_006270 | T |
| Bacillus subtilis subsp. subtilis str. 168 | NC_000964 | T |
| Bacillus thuringiensis serovar konkukian str. 97-27 | NC_005957 | T |
| Bacillus weihenstephanensis KBAB4 | NC_010184 | T |
| Listeria innocua Clip11262 | NC_003212 | T |
| Listeria monocytogenes EGD-e | NC_003210 | T |
| Listeria welshimeri serovar 6b str. SLCC5334 | NC_008555 | T |
| Moorella thermoacetica ATCC 39073 | NC_007644 | T |
| Chloroflexus aurantiacus J-10-fl | NC_010175 | T |
| Dehalococcoides ethenogenes 195 | NC_002936 | T |
| Dehalococcoides sp. BAV1 | NC_009455 | T |
| Dehalococcoides sp. CBDB1 | NC_007356 | T |
| Herpetosiphon aurantiacus ATCC 23779 | NC_009972 | F |
| Roseiflexus castenholzii DSM 13941 | NC_009767 | T |
| Roseiflexus sp. RS-1 | NC_009523 | T |
| Gloeobacter violaceus PCC 7421 | NC_005125 | T |
| Prochlorococcus marinus subsp. marinus str. CCMP1375 | NC_005042 | F |
| Synechococcus elongatus PCC 6301 | NC_006576 | T |
| Synechococcus sp. CC9311 | NC_008319 | T |

Table S2. *(Continued)*

| Bacteria name | NCBI accession No. | T/F |
|---|---|---|
| Synechococcus sp. CC9605 | NC_007516 | T |
| Synechococcus sp. CC9902 | NC_007513 | T |
| Synechococcus sp. JA-2-3B'a(2-13) | NC_007776 | T |
| Synechococcus sp. RCC307 | NC_009482 | T |
| Synechococcus sp. WH 7803 | NC_009481 | T |
| Synechococcus sp. WH 8102 | NC_005070 | T |
| Synechocystis sp. PCC 6803 | NC_000911 | T |
| Thermosynechococcus elongatus BP-1 | NC_004113 | T |
| Trichodesmium erythraeum IMS101 | NC_008312 | T |
| Bifidobacterium adolescentis ATCC 15703 | NC_008618 | T |
| Clavibacter michiganensis subsp. michiganensis NCPPB 382 | NC_009480 | T |
| Corynebacterium efficiens YS-314 | NC_004369 | T |
| Corynebacterium glutamicum ATCC 13032 | NC_003450 | T |
| Corynebacterium jeikeium K411 | NC_007164 | T |
| Frankia alni ACN14a | NC_008278 | T |
| Frankia sp. EAN1pec | NC_009921 | T |
| Kineococcus radiotolerans SRS30216 | NC_009664 | T |
| Leifsonia xyli subsp. xyli str. CTCB07 | NC_006087 | T |
| Propionibacterium acnes KPA171202 | NC_006085 | T |
| Renibacterium salmoninarum ATCC 33209 | NC_010168 | T |
| Rhodococcus jostii RHA1 | NC_008268 | T |
| Saccharopolyspora erythraea NRRL 2338 | NC_009142 | T |
| Salinispora arenicola CNS-205 | NC_009953 | T |
| Salinispora tropica CNB-440 | NC_009380 | T |
| Streptococcus suis 05ZYH33 | NC_009442 | F |
| Streptomyces avermitilis MA-4680 | NC_003155 | T |

Table S2. *(Continued)*

| Bacteria name | NCBI accession No. | T/F |
|---|---|---|
| Streptomyces coelicolor A3(2) | NC_003888 | T |
| Thermobifida fusca YX | NC_007333 | T |
| Tropheryma whipplei TW08/27 | NC_004551 | T |
| Acidothermus cellulolyticus 11B | NC_008578 | T |
| Arthrobacter sp. FB24 | NC_008541 | T |
| Mycobacterium avium subsp. paratuberculosis K-10 | NC_002944 | T |
| Mycobacterium bovis AF2122/97 | NC_002945 | T |
| Mycobacterium gilvum PYR-GCK | NC_009338 | T |
| Mycobacterium leprae TN | NC_002677 | T |
| Mycobacterium smegmatis str. MC2 155 | NC_008596 | T |
| Mycobacterium sp. JLS | NC_009077 | T |
| Mycobacterium sp. KMS | NC_008705 | T |
| Mycobacterium sp. MCS | NC_008146 | T |
| Mycobacterium tuberculosis H37Rv | NC_000962 | T |
| Mycobacterium ulcerans Agy99 | NC_008611 | F |
| Mycobacterium vanbaalenii PYR-1 | NC_008726 | T |
| Pyrobaculum aerophilum IM2 | NC_003364 | F |
| Sulfolobus solfataricus P2 | NC_002754 | F |
| Aeropyrum pernix K1 | NC_000854 | F |
| Halobacterium salinarium NRC-1 | NC_002607 | F |
| Pyrococcus horikoshii OT3 | NC_000961 | F |
| Thermoplasma acidophilum DSM 1728 | NC_002578 | F |
| Archaeoglobus fulgidus DSM 4304 | NC_000917 | F |
| Methanococcus jannaschii DSM 2661 | NC_000909 | F |
| Nitrosopumilus maritimus SCM1 | NC_010085 | F |
