# Supplementary material for: A Preliminary Metagenome Analysis Based on a Combination of Protein Domains
Source: Proteomes. 2019 Apr 29;7(2):19. doi: 10.3390/proteomes7020019 (PMC6630717; doi:10.3390/proteomes7020019)
Supplement: Supplementary file 1 [file proteomes-07-00019-s001.zip › supplementary/Table S3.pdf]

Table S3. Percentage of protein_ids picked-up for respective species
T: True (picked-up); F: False (not picked-up)

NC Number	T	F	Ratio (%)
NC_008278	5398	375	93.5
NC_008536	7257	464	94.1
NC_009921	6639	312	95.5
NC_008312	4222	165	96.2
NC_009379	2057	58	97.3
NC_003155	7181	162	97.8
NC_002678	6452	105	98.4
NC_009445	6440	77	98.8
NC_008095	7069	74	99
NC_008268	6827	41	99.4
NC_008380	4731	20	99.6
NC_009648	5041	6	99.9
NC_000117	887	0	100
NC_000853	1858	0	100
NC_000907	1610	0	100
NC_000911	3230	0	100
NC_000913	4140	0	100
NC_000915	1445	0	100
NC_000918	1497	0	100
NC_000919	970	0	100
