# Supplementary material for: A Preliminary Metagenome Analysis Based on a Combination of Protein Domains
Source: Proteomes. 2019 Apr 29;7(2):19. doi: 10.3390/proteomes7020019 (PMC6630717; doi:10.3390/proteomes7020019)
Supplement: Supplementary file 1 [file proteomes-07-00019-s001.zip › supplementary/Table S1.pdf]

Table S1. List of names and NCBI accession numbers of bacteria used in present analyses

Bacteria name	NCBI accession No.
Fervidobacterium nodosum Rt17-B1	NC_009718
Petrotoga mobilis SJ95	NC_010003
Thermosipho melanesiensis BI429	NC_009616
Thermotoga lettingae TMO	NC_009828
Thermotoga maritima MSB8	NC_000853
Thermotoga petrophila RKU-1	NC_009486
Candidus ‘Acertothermus autotrophicum	AP011800-03
Deinococcus geothermalis DSM 11300	NC_008025
Deinococcus radiodurans R1	NC_001263-64
Thermus 1longates1es HB27	NC_005835
Borrelia afzelii Pko	NC_008277
Borrelia burgdorferi B31	NC_001318
Borrelia garinii Pbi	NC_006156
Leptospira borgpetersenii serovar Hardjo-bovis JB197	NC_008510-11
Treponema denticola ATCC 35405	NC_002967
Treponema pallidumsub sp. Pallidum str. Nichols	NC_000919
Leptospira interrogans serovar Lai str. 56601	NC_004342-43
Chlorobium chlorochromatii CaD3	NC_007514
Chlorobium phaeobacteroides DSM 266	NC_008639
Chlorobium tepidum TLS	NC_002932
Pelodictyon luteolum DSM 273	NC_007512
Prosthecochloris vibrioformis DSM 265	NC_009337
Cytophaga hutchinsonii ATCC 33406	NC_008255
Flavobacterium johnsoniae UW101	NC_009441
Gramella forsetii KT0803	NC_008571

Table S1. *(Continued)*

Bacteria name	NCBI accession No.
Porphyromonas gingivalis W83	NC_002950
Salinibacter ruber DSM 13855	NC_007677
Bacteroides fragilis YCH46	NC_006347
Bacteroides thetaiotaomicron VPI-5482	NC_004663
Rhodopirellula baltica SH 1	NC_005027
Candidatus Protochlamydia amoebophila UWE25	NC_005861
Chlamydia muridarum Nigg	NC_002620
Chlamydia trachomatis D/UW-3/CX	NC_000117
Chlamydophila abortus S26/3	NC_004552
Chlamydophila caviae GPIC	NC_003361
Chlamydophila pneumoniae CWL029	NC_000922
Aquifex aeolicus VF5	NC_000918
Arcobacter butzleri RM4018	NC_009850
Campylobacter fetus subsp. Fetus 82-40	NC_008599
Campylobacter jejuni subsp. Jejuni NCTC 11168	NC_002163
Helicobacter hepaticus ATCC 51449	NC_004917
Helicobacter pylori 26695	NC_000915
Sulfurimonas denitrificans DSM 1251	NC_007575
Sulfurovum sp. NBC37-1	NC_009663
Wolinella succinogenes DSM 1740	NC_005090
Nitratiruptor sp. SB155-2	NC_009662
Anaeromyxobacter sp. Fw109-5	NC_009675
Bdellovibrio bacteriovorus HD100	NC_005363
Desulfococcus oleovorans Hxd3	NC_009943
Desulfotalea psychrophila LSv54	NC_006138
Desulfovibrio desulfuricans G20	NC_007519
Desulfovibrio vulgaris subsp. Vulgaris str. Hildenborough	NC_002937

Table S1. *(Continued)*

Bacteria name	NCBI accession No.
Geobacter metallireducens GS-15	NC_007517
Geobacter sulfurreducens PCA	NC_002939
Geobacter uraniireducens Rf4	NC_009483
Pelobacter carbinolicus DSM 2380	NC_007498
Pelobacter propionicus DSM 2379	NC_008609
Sorangium cellulosum ‘So ce 56’	NC_010162
Syntrophobacter fumaroxidans MPOB	NC_008554
Syntrophus aciditrophicus SB	NC_007759
Myxococcus xanthus DK 1622	NC_008095
Magnetococcus sp. MC-1	NC_008576
Bradyrhizobium japonicum USDA110	NC_004463
Bradyrhizobium sp. ORS278	NC_009445
Brucella abortus biovar 1 str. 9-941	NC_006932-33
Brucella canis ATCC 23365	NC_010103-104
Brucella melitensis 16M	NC_003317-18
Brucella ovis ATCC 25840	NC_009504-05
Brucella suis 1330	NC_004310-11
Candidatus Pelagibacter ubique HTCC1062	NC_007205
Caulobacter crescentus CB15	NC_002696
Dinoroseobacter shibae DFL 12	NC_009952
Erythrobacter litoralis HTCC2594	NC_007722
Gluconacetobacter diazotrophicus Pal 5	NC_010125
Gluconobacter oxydans 621H	NC_006677
Granulobacter bethesdensis CGDNIH1	NC_008343
Hyphomonas neptunium ATCC 15444	NC_008358
Jannaschia sp. CCS1	NC_007802
Ochrobactrum 3longates ATCC 49188	NC_009667-68

Table S1. *(Continued)*

Bacteria name	NCBI accession No.
Parvibaculum lavamentivorans DS-1	NC_009719
Rhizobium etli CFN 42	NC_007761
Rhizobium leguminosarum bv.viciae 3841	NC_008380
Rhodobacter sphaeroides 2.4.1	NC_007493-94
Rhodopseudomonas palustris CGA009	NC_005296
Rhodospirillum rubrum ATCC 11170	NC_007643
Roseobacter denitrificans Och 114	NC_008209
Rubrobacter xylanophilus DSM 9941	NC_008148
Silicibacter pomeroyi DSS-3	NC_003911
Silicibacter sp. TM1040	NC_008044
Sinorhizobium medicae WSM419	NC_009636
Sinorhizobium meliloti 1021	NC_003047
Sphingomonas wittichii RW1	NC_009511
Sphingopyxis alaskensis RB2256	NC_008048
Wolbachia endosymbiont of Drosophila melanogaster	NC_002978
Wolbachia endosymbiont strain TRS of Brugia malayi	NC_006833
Xanthobacter autotrophicus Py2	NC_009720
Zymomonas mobilis subsp. Mobilis ZM4	NC_006526
Magnetospirillum magneticum AMB-1	NC_007626
Maricaulis maris MCS10	NC_008347
Mesorhizobium loti MAFF303099	NC_002678
Mesorhizobium sp. BNC1	NC_008254
Methylobacterium extorquens PA1	NC_010172
Neorickettsia sennetsu str. Miyayama	NC_007798
Nitrobacter hamburgensis X14	NC_007964
Nitrobacter winogradskyi Nb-255	NC_007406
Acidiphilium cryptum JF-5	NC_009484

Table S1. *(Continued)*

Bacteria name	NCBI accession No.
Agrobacterium tumefaciens str. C58	NC_003062-63
Anaplasma marginale str. St. Maries	NC_004842
Anaplasma phagocytophilum HZ	NC_007797
Azorhizobium caulinodans ORS 571	NC_009937
Bartonella bacilliformis KC583	NC_008783
Bartonella henselae str. Houston-1	NC_005956
Bartonella 5longate str. Toulouse	NC_005955
Bartonella tribocorum CIP 105476	NC_010161
Acidovorax avenae subsp. Citrulli AAC00-1	NC_008752
Acidovorax sp. JS42	NC_008782
Azoarcus sp. BH72	NC_008702
Azoarcus sp. EbN1	NC_006513
Bordetella bronchiseptica RB50	NC_002927
Bordetella parapertussis 12822	NC_002928
Bordetella pertussis Tohama I	NC_002929
Bordetella petrii DSM 12804	NC_010170
Burkholderia ambifaria AMMD	NC_010551-52,57
Burkholderia cenocepacia AU 1054	NC_008060-62
Burkholderia multivorans ATCC 17616	NC_010086-87. 84
Burkholderia pseudomallei K96243	NC_006350-51
Burkholderia sp. 383	NC_007509-11
Burkholderia thailandensis E264	NC_007650-51
Burkholderia vietnamiensis G4	NC_009254-56
Burkholderia xenovorans LB400	NC_007951-53
Chromobacterium violaceum ATCC 12472	NC_005085
Dechloromonas 5longate RCB	NC_007298
Delftia acidovorans SPH-1	NC_010002

Table S1. *(Continued)*

Bacteria name	NCBI accession No.
Nitrosomonas europaea ATCC 19718	NC_004757
Nitrosomonas eutropha C91	NC_008344
Nitrosospira multiformis ATCC 25196	NC_007614
Polaromonas sp. JS666	NC_007948
Polynucleobacter sp. QLW-P1DMWA-1	NC_009379
Ralstonia solanacearum GMI1000	NC_003295
Rhodoferax ferrireducens T118	NC_007908
Thiobacillus denitrificans ATCC 25259	NC_007404
Verminephrobacter eiseniae EF01-2	NC_008786
Methylobacillus 6longates6e KT	NC_007947
Neisseria gonorrhoeae FA 1090	NC_002946
Neisseria 6longates6es MC58	NC_002946
Acinetobacter sp. ADP1	NC_005966
Actinobacillus pleuropneumoniae L20	NC_009053
Actinobacillus succinogenes 130Z	NC_009655
Aeromonas hydrophila subsp. Hydrophila ATCC 7966	NC_008570
Aeromonas salmonicida subsp. Salmonicida A449	NC_009348
Alcanivorax borkumensis SK2	NC_008260
Alkalilimnicola ehrlichei MLHE-1	NC_008340
Baumannia cicadellinicola str. Hc (Homalodisca 6longates)	NC_007984
Buchnera aphidicola str. APS (Acyrthosiphon pisum)	NC_002528
Candidatus Blochmannia floridanus	NC_005061
Candidatus Blochmannia pennsylvanicus str. BPEN	NC_007292
Candidatus Ruthia magnifica str. Cm (Calyptogena magnifica)	NC_008610
Candidatus Vesicomyosocius okutanii HA	NC_009465
Chromohalobacter salexigens DSM 3043	NC_007963

Table S1. *(Continued)*

Bacteria name	NCBI accession No.
Citrobacter koseri ATCC BAA-895	NC_009792
Colwellia psychrerythraea 34H	NC_003910
Coxiella burnetii RSA 493	NC_002971
Ehrlichia canis str. Jake	NC_007354
Ehrlichia chaffeensis str. Arkansas	NC_007799
Ehrlichia ruminantium str. Welgevonden	NC_005295
Enterobacter sakazakii ATCC BAA-894	NC_009778
Enterobacter sp. 638	NC_009436
Erwinia carotovora subsp. Atroseptica SCRI1043	NC_004547
Escherichia coli K-12 MG1655	NC_000913
Francisella tularensis subsp. Novicida U112	NC_008601
Francisella tularensis subsp. Tularensis SCHU S4	NC_006570
Haemophilus ducreyi 35000HP	NC_002940
Haemophilus 7longates Rd KW20	NC_000907
Haemophilus somnus 129PT	NC_008309
Hahella chejuensis KCTC 2396	NC_007645
Halorhodospira 7longates SL1	NC_008789
Idiomarina loihiensis L2TR	NC_006512
Klebsiella pneumoniae subsp. Pneumoniae MGH 78578	NC_009648
Legionella pneumophila subsp. Pneumophila str. Philadelphia 1	NC_002942
Pasteurella multocida subsp. Multocidastr. Pm70	NC_002663
Photobacterium profundum SS9	NC_006370-71
Photorhabdus luminescens subsp. Laumondii TTO1	NC_005126
Pseudoalteromonas atlantica T6c	NC_008228
Pseudoalteromonas haloplanktis TAC125	NC_007481-82
Pseudomonas aeruginosa PAO1	NC_002516

Table S1. *(Continued)*

Bacteria name	NCBI accession No.
Pseudomonas entomophila L48	NC_008027
Pseudomonas fluorescens Pf-5	NC_004129
Pseudomonas mendocina ymp	NC_009439
Pseudomonas putida KT2440	NC_002947
Pseudomonas stutzeri A1501	NC_009434
Pseudomonas syringae pv. Phaseolicola 1448A	NC_005773
Pseudomonas syringae pv. Syringae B728a	NC_007005
Pseudomonas syringae pv. Tomato str. DC3000	NC_004578
Psychrobacter arcticus 273-4	NC_007204
Psychrobacter cryohalolentis K5	NC_007969
Psychrobacter sp. PRwf-1	NC_009524
Psychromonas ingrahamii 37	NC_008709
Saccharophagus degradans 2-40	NC_007912
Salmonella typhimurium LT2	NC_003197
Serratia proteamaculans 568	NC_009832
Shewanella amazonensis SB2B	NC_008700
Shewanella denitrificans OS217	NC_007954
Shewanella frigidimarina NCIMB 400	NC_008345
Shewanella loihica PV-4	NC_009092
Shewanella oneidensis MR-1	NC_004347
Shewanella pealeana ATCC 700345	NC_009901
Shewanella putrefaciens CN-32	NC_009438
Shewanella sediminis HAW-EB3	NC_009831
Shewanella sp. ANA-3	NC_008577
Shewanella sp. MR-4	NC_008321
Shewanella sp. MR-7	NC_008322
Shewanella sp. W3-18-1	NC_008750

Table S1. *(Continued)*

Bacteria name	NCBI accession No.
Shigella boydii Sb227	NC_007613
Shigella dysenteriae Sd197	NC_007606
Shigella flexneri 2a str. 301	NC_004337
Shigella sonnei Ss046	NC_007384
Thiomicrospira crunogena XCL-2	NC_007520
Vibrio 9longat O1 biovar eltor str. N16961	NC_002505-06
Vibrio fischeri ES114	NC_006840-41
Vibrio harveyi ATCC BAA-1116	NC_009783-84
Vibrio vulnificus CMCP6	NC_004459-60
Wigglesworthia glossinidia endosymbiont of Glossina brevipalpis	NC_004344
Xanthomonas axonopodis pv. Citristr. 306	NC_003919
Xanthomonas campestris pv. Campestris str. ATCC 33913	NC_007086
Xanthomonas campestris pv. Vesicatoria str. 85-10	NC_007508
Xanthomonas oryzae pv. Oryzae KACC10331	NC_006834
Xylella fastidiosa 9a5c	NC_002488
Yersinia enterocolitica subsp. Enterocolitica 8081	NC_008800,NC_008791
Yersinia pestis CO92	NC_003143
Yersinia pseudotuberculosis IP 32953	NC_006155
Mannheimia succiniciproducens MBEL55E	NC_006300
Marinobacter aquaeolei VT8	NC_008740
Marinomonas sp. MWYL1	NC_009654
Nitrosococcus oceani ATCC 19707	NC_007484
Solibacter usitatus Ellin6076	NC_008536
Acidobacteria bacterium Ellin345	NC_008009
Onion yellows phytoplasma OY-M	NC_005303
Ureaplasma parvumserovar 3 str. ATCC 700970	NC_002162

Table S1. *(Continued)*

Bacteria name	NCBI accession No.
Acholeplasma laidlawii PG-8A	NC_010163
Aster yellows witches'-broom phytoplasma AYWB	NC_007716
Mesoplasma florum L1	NC_006055
Mycoplasma agalactiae PG2	NC_009497
Mycoplasma capricolum subsp. Capricolum ATCC 27343	NC_007633
Mycoplasma gallisepticum R	NC_004829
Mycoplasma hyopneumoniae 232	NC_006360
Mycoplasma mobile 163K	NC_006908
Mycoplasma mycoides subsp. Mycoides SC str. PG1	NC_005364
Mycoplasma penetrans HF-2	NC_004432
Caldicellulosiruptor saccharolyticus DSM 8903	NC_009437
Carboxydothermus hydrogenoformans Z-2901	NC_007503
Clostridium acetobutylicum ATCC 824	NC_003030
Clostridium beijerinckii NCIMB 8052	NC_009617
Clostridium botulinum A str. ATCC 3502	NC_009495
Clostridium kluyveri DSM 555	NC_009706
Clostridium novyi NT	NC_008593
Clostridium perfringens str. 13	NC_003366
Clostridium phytofermentans ISDg	NC_010001
Clostridium thermocellum ATCC 27405	NC_009012
Desulfitobacterium hafniense Y51	NC_007907
Desulfotomaculum reducens MI-1	NC_009253
Enterococcus faecalis V583	NC_004668
Geobacillus kaustophilus HTA426	NC_006510
Geobacillus thermodenitrificans NG80-2	NC_009328
Lactobacillus acidophilus NCFM	NC_006814
Lactobacillus casei ATCC 334	NC_008526

Table S1. *(Continued)*

Bacteria name	NCBI accession No.
Lactobacillus delbrueckii subsp. Bulgaricus ATCC 11842	NC_008054
Lactobacillus gasseri ATCC 33323	NC_008530
Lactobacillus helveticus DPC 4571	NC_010080
Lactobacillus johnsonii NCC 533	NC_005362
Lactobacillus plantarum WCFS1	NC_004567
Lactobacillus sakei subsp. Sakei 23K	NC_007576
Lactobacillus salivarius UCC118	NC_007929
Lactococcus lactis subsp. Lactis IL1403	NC_002662
Oceanobacillus iheyensis CAN831	NC_004193
Oenococcus oeni PSU-1	NC_008528
Pediococcus pentosaceus ATCC 25745	NC_008525
Pelotomaculum thermopropionicum SI	NC_009454
Staphylococcus aureus subsp. Aureus N315	NC_002745
Staphylococcus epidermidis ATCC 12228	NC_004461
Staphylococcus haemolyticus JCSC1435	NC_007168
Staphylococcus saprophyticus subsp. Saprophyticus ATCC 15305	NC_007350
Streptococcus agalactiae 2603V/R	NC_004116
Streptococcus gordonii str. Challis substr. CH1	NC_009785
Streptococcus mutans UA159	NC_013928
Streptococcus pneumoniae TIGR4	NC_003028
Streptococcus pyogenes M1 GAS	NC_002737
Streptococcus sanguinis SK36	NC_009009
Streptococcus 11longates11es CNRZ1066	NC_006449
Symbiobacterium thermophilum IAM 14863	NC_006177
Syntrophomonas wolfei subsp. Wolfeistr. Goettingen	NC_008346
Thermoanaerobacter ethanolicus ATCC 33223	NC_010321

Table S1. *(Continued)*

Bacteria name	NCBI accession No.
Thermoanaerobacter ethanolicus X514	NC_010320
Thermoanaerobacter tengcongensis MB4	NC_003869
Alkaliphilus metalliredigens QYMF	NC_009633
Bacillus amyloliquefaciens FZB42	NC_009725
Bacillus anthracis str. Ames	NC_003997
Bacillus clausii KSM-K16	NC_006582
Bacillus licheniformis ATCC 14580	NC_006270
Bacillus subtilis subsp. Subtilis str. 168	NC_000964
Bacillus thuringiensis serovar konkukian str. 97-27	NC_005957
Bacillus weihenstephanensis KBAB4	NC_010184
Listeria innocua Clip11262	NC_003212
Listeria monocytogenes EGD-e	NC_003210
Listeria welshimeri serovar 6b str. SLCC5334	NC_008555
Moorella thermoacetica ATCC 39073	NC_007644
Chloroflexus aurantiacus J-10-fl	NC_010175
Dehalococcoides ethenogenes 195	NC_002936
Dehalococcoides sp. BAV1	NC_009455
Dehalococcoides sp. CBDB1	NC_007356
Herpetosiphon aurantiacus ATCC 23779	NC_009972
Roseiflexus castenholzii DSM 13941	NC_009767
Roseiflexus sp. RS-1	NC_009523
Gloeobacter violaceus PCC 7421	NC_005125
Prochlorococcus marinus subsp. Marinus str. CCMP1375	NC_005042
Synechococcus 12longates PCC 6301	NC_006576
Synechococcus sp. CC9311	NC_008319
Synechococcus sp. CC9605	NC_007516
Synechococcus sp. CC9902	NC_007513

Table S1. *(Continued)*

Bacteria name	NCBI accession No.
Synechococcus sp. JA-2-3B'a(2-13)	NC_007776
Synechococcus sp. RCC307	NC_009482
Synechococcus sp. WH 7803	NC_009481
Synechococcus sp. WH 8102	NC_005070
Synechocystis sp. PCC 6803	NC_000911
Thermosynechococcus 13longates BP-1	NC_004113
Trichodesmium erythraeum IMS101	NC_008312
Bifidobacterium adolescentis ATCC 15703	NC_008618
Clavibacter michiganensis subsp. Michiganensis NCPPB 382	NC_009480
Corynebacterium efficiens YS-314	NC_004369
Corynebacterium glutamicum ATCC 13032	NC_003450
Corynebacterium jeikeium K411	NC_007164
Frankia alni CAN14a	NC_008278
Frankia sp. EAN1pec	NC_009921
Kineococcus radiotolerans SRS30216	NC_009664
Leifsonia xyli subsp. Xyli str. CTCB07	NC_006087
Propionibacterium acnes KPA171202	NC_006085
Renibacterium salmoninarum ATCC 33209	NC_010168
Rhodococcus jostii RHA1	NC_008268
Saccharopolyspora erythraea NRRL 2338	NC_009142
Salinispora arenicola CNS-205	NC_009953
Salinispora tropica CNB-440	NC_009380
Streptococcus suis 05ZYH33	NC_009442
Streptomyces avermitilis MA-4680	NC_003155
Streptomyces coelicolor A3(2)	NC_003888
Thermobifida fusca YX	NC_007333

Table S1. *(Continued)*

Bacteria name	NCBI accession No.
Tropheryma whipplei TW08/27	NC_004551
Acidothermus cellulolyticus 11B	NC_008578
Arthrobacter sp. FB24	NC_008541
Mycobacterium avium subsp. Paratuberculosis K-10	NC_002944
Mycobacterium bovis AF2122/97	NC_002945
Mycobacterium gilvum PYR-GCK	NC_009338
Mycobacterium leprae TN	NC_002677
Mycobacterium smegmatis str. MC2 155	NC_008596
Mycobacterium sp. JLS	NC_009077
Mycobacterium sp. KMS	NC_008705
Mycobacterium sp. MCS	NC_008146
Mycobacterium tuberculosis H37Rv	NC_000962
Mycobacterium ulcerans Agy99	NC_008611
Mycobacterium vanbaalenii PYR-1	NC_008726
Pyrobaculum aerophilum IM2	NC_003364
Sulfolobus solfataricus P2	NC_002754
Aeropyrum pernix K1	NC_000854
Halobacterium salinarium NRC-1	NC_002607
Pyrococcus horikoshii OT3	NC_000961
Thermoplasma acidophilum DSM 1728	NC_002578
Archaeoglobus fulgidus DSM 4304	NC_000917
Methanococcus jannaschii DSM 2661	NC_000909
Nitrosopumilus maritimus SCM1	NC_010085

*
