# Supplementary material for: A Preliminary Metagenome Analysis Based on a Combination of Protein Domains
Source: Proteomes. 2019 Apr 29;7(2):19. doi: 10.3390/proteomes7020019 (PMC6630717; doi:10.3390/proteomes7020019)
Supplement: Supplementary file 1 [file proteomes-07-00019-s001.zip › supplementary/Table S4.pdf]

Table S4. Proportions of coding regions of bacteria analyzed

The rightmost column shows the ratio (%).

NC Number	Amino acid	DNA	(Amino*3/DNA)*100
NC_005070	22334	2434428	2.75226870542
NC_002677	538412	3268203	49.4227561752
NC_008312	1425008	7750108	55.1608313071
NC_005295	314598	1516355	62.2409660007
NC_007716	154728	706569	65.6954947075
NC_005303	187071	853092	65.7857534709
NC_006833	238295	1080084	66.1879076072
NC_007797	330732	1471282	67.4375136785
NC_007354	313302	1315030	71.4741108568
NC_005956	460701	1931047	71.5727271268
NC_006087	625832	2584158	72.6540714616
NC_007606	1063403	4369232	73.0153262633
NC_005955	385163	1581384	73.0682111366
NC_010161	638669	2619061	73.1562571471
NC_008278	1880148	7497934	75.2266424324
NC_009921	2256560	8982042	75.3690530505
NC_006834	1242874	4941439	75.4561980832
NC_007292	199482	791654	75.5943884576
NC_010080	525431	2080931	75.7494121622
NC_010168	799011	3155250	75.9696695983
NC_008054	473533	1864998	76.1716098355
NC_002946	549846	2153922	76.5829960416
NC_007799	300662	1176248	76.6833184839
NC_002488	686608	2679306	76.8790126996
NC_008709	1172297	4559598	77.1316023913
NC_002978	325983	1267782	77.1385774526

Table S4. *(Continued)*

NC Number	Amino acid	DNA	(Amino*3/DNA)*100
NC_006570	490394	1892775	77.7261956651
NC_004337	1195937	4607202	77.8739677574
NC_002971	519611	1995281	78.1259882693
NC_006449	468987	1796226	78.3287292356
NC_009617	1573936	6000632	78.6885114768
NC_007964	1164089	4406967	79.2442285136
NC_005861	638604	2414465	79.3472674071
NC_009497	232692	877438	79.5584417361
NC_008610	308184	1160782	79.6490641654
NC_003143	1235755	4653728	79.6622621692
NC_005126	1512057	5688987	79.7360057247
NC_007613	1201924	4519823	79.7768408188
NC_008783	384545	1445021	79.8351719456
NC_009348	1255231	4702402	80.0802015651
NC_003155	2412239	9025608	80.1798283285
NC_010175	1406407	5258541	80.2355824553
NC_007204	709239	2650701	80.2699738673
NC_003997	1406113	5227293	80.6983461612
NC_009495	1045624	3886916	80.703364827
NC_008554	1342612	4990251	80.7140963451
NC_010001	1304282	4847594	80.7172795411
NC_002940	457468	1698955	80.7793025713
NC_007384	1301481	4825265	80.9166543185
NC_003366	820130	3031430	81.1626855972
NC_007350	681369	2516575	81.2257532559
NC_009012	1041550	3843301	81.3012043553

Table S4. *(Continued)*

NC Number	Amino acid	DNA	(Amino*3/DNA)*100
NC_005364	329650	1211703	81.6165347449
NC_009767	1559762	5723298	81.7585594879
NC_010172	1491310	5471154	81.773059212
NC_004347	1355450	4969811	81.8210189482
NC_007508	1412578	5178466	81.8337708503
NC_010184	1435908	5262775	81.8527107847
NC_006138	963050	3523383	81.9993171336
NC_009524	814717	2978976	82.0466831556
NC_008526	792434	2895264	82.1100251998
NC_005773	1623377	5928787	82.1438010844
NC_008528	487641	1780517	82.162821248
NC_009633	1350595	4929566	82.1935440158
NC_009328	973070	3550319	82.2238790373
NC_002929	1120083	4086189	82.2343019376
NC_008346	806024	2936195	82.3539308527
NC_006155	1303152	4744671	82.396777353
NC_007333	1000848	3642249	82.4365385233
NC_009831	1516677	5517674	82.4628457571
NC_007406	935366	3402093	82.4815194646
NC_006510	975239	3544776	82.5360192012
NC_005061	194117	705557	82.5377680329
NC_007969	842792	3059876	82.6300150725
NC_008345	1337797	4845257	82.8313338178
NC_002950	647056	2343476	82.8328517126
NC_003910	1485044	5373180	82.9142518955
NC_009901	1430865	5174581	82.9554122353
NC_007954	1257435	4545906	82.9824681813

Table S4. *(Continued)*

NC Number	Amino acid	DNA	(Amino*3/DNA)*100
NC_009706	1097282	3964618	83.0305971471
NC_004461	692957	2499279	83.1788287742
NC_009445	2067579	7456587	83.1846661214
NC_009454	839227	3025375	83.2188075858
NC_004567	917837	3308273	83.2310695036
NC_002162	208768	751719	83.3162391798
NC_004842	332640	1197687	83.3206004574
NC_007168	745871	2685015	83.3370763292
NC_009253	1002759	3608104	83.3755623452
NC_004193	1009149	3630528	83.3886145486
NC_008639	871247	3133902	83.4021293582
NC_006177	991447	3566135	83.4051711447
NC_007086	1431716	5148708	83.4218603968
NC_007516	698228	2510659	83.431640856
NC_000907	508974	1830138	83.4320690571
NC_009437	826176	2970275	83.4443948793
NC_007614	886770	3184243	83.546073588
NC_002737	515984	1852433	83.5631842015
NC_003919	1442184	5175554	83.5959203594
NC_007776	849163	3046682	83.615191871
NC_005957	1459845	5237682	83.6159010799
NC_002745	784685	2814816	83.6308661028
NC_009523	1618449	5801598	83.6898213216
NC_003028	602939	2160842	83.7088968097
NC_004547	1414406	5064019	83.7915102609
NC_002678	1966772	7036071	83.8581077422
NC_002662	661258	2365589	83.8596222759

Table S4. *(Continued)*

NC Number	Amino acid	DNA	(Amino*3/DNA)*100
NC_007929	510776	1827111	83.8661690505
NC_009142	2301348	8212805	84.0643848235
NC_007984	192742	686194	84.2656741388
NC_008786	1563779	5566749	84.2742685183
NC_010162	3661795	13033779	84.283959395
NC_007576	529500	1884661	84.2857150437
NC_009953	1626445	5786361	84.324759551
NC_007907	1610231	5727534	84.3415857505
NC_007798	241766	859006	84.4345673953
NC_008618	588175	2089645	84.441376406
NC_007498	1031868	3665893	84.4433811898
NC_008536	2809575	9965640	84.5778595253
NC_008578	689147	2443540	84.6084369398
NC_007677	1002053	3551823	84.6370722865
NC_009438	1314712	4659220	84.6522808539
NC_009720	1498967	5308934	84.7044058186
NC_013928	568632	2013587	84.7192597092
NC_007759	898142	3179300	84.749032806
NC_008322	1354130	4792610	84.7636256653
NC_000913	1312010	4641652	84.7980417317
NC_010125	1115229	3944163	84.8262863376
NC_009092	1301417	4602594	84.8271865822
NC_008344	752727	2661057	84.8603017523
NC_008750	1332575	4708380	84.9065920763
NC_008576	1335859	4719581	84.9138302743
NC_003295	1051971	3716413	84.9182531651
NC_009465	289450	1022154	84.9529522949

Table S4. *(Continued)*

NC Number	Amino acid	DNA	(Amino*3/DNA)*100
NC_003030	1115965	3940880	84.9529800451
NC_007761	1241001	4381608	84.9688744406
NC_006582	1220192	4303871	85.0531068427
NC_008319	739449	2606748	85.1001707875
NC_002928	1354157	4773551	85.1037519029
NC_004578	1815796	6397126	85.153676823
NC_008577	1411737	4972204	85.1777400927
NC_008593	723920	2547720	85.2432763412
NC_008752	1521936	5352772	85.2980100778
NC_009053	646731	2274482	85.3026315442
NC_006677	768489	2702173	85.3190006709
NC_007484	990226	3481691	85.3228503046
NC_008321	1338772	4706287	85.3393768803
NC_003869	765819	2689445	85.42494827
NC_007912	1441037	5057531	85.4786851529
NC_005085	1354078	4751080	85.5012754995
NC_000915	475362	1667867	85.5035803215
NC_004551	264091	925938	85.5643682406
NC_009380	1478492	5183331	85.5719227655
NC_008228	1480845	5187005	85.6474015352
NC_008380	1443995	5057142	85.6607348578
NC_009483	1466699	5136364	85.6655992449
NC_003450	945250	3309401	85.6877120663
NC_009778	1247885	4368373	85.6990691958
NC_008609	1145502	4008000	85.7411676647
NC_005362	570109	1992676	85.8306618838
NC_010320	703108	2457259	85.8405239334

Table S4. *(Continued)*

NC Number	Amino acid	DNA	(Amino*3/DNA)*100
NC_006085	732749	2560265	85.8601355719
NC_004668	921034	3218031	85.8631256194
NC_007644	752407	2628784	85.8655941302
NC_007633	289149	1010023	85.8838858125
NC_006526	588775	2056363	85.8955836105
NC_010321	677415	2362816	86.0094480484
NC_008782	1275648	4448856	86.0208556986
NC_005296	1565515	5459213	86.0297079451
NC_007645	2069164	7215267	86.0327414079
NC_009937	1540134	5369772	86.0446588794
NC_003047	1048533	3654135	86.0832727855
NC_007519	1070627	3730232	86.104054654
NC_000911	1026816	3573470	86.2032702107
NC_007948	1494587	5200264	86.2217956627
NC_006270	1213919	4222597	86.2444841409
NC_002937	1026885	3570858	86.2721228343
NC_006513	1235661	4296230	86.28455646
NC_004116	621357	2160267	86.2889170644
NC_003197	1397356	4857432	86.302144837
NC_006300	665751	2314078	86.3088020369
NC_004757	809267	2812094	86.3342761657
NC_008260	899628	3120143	86.4987277827
NC_004829	292046	1012800	86.5065165877
NC_008268	2254683	7804765	86.66563311
NC_004344	201566	697724	86.6672208495
NC_009832	1574464	5448853	86.6859869407
NC_002528	185191	640681	86.7160099956

Table S4. *(Continued)*

NC Number	Amino acid	DNA	(Amino*3/DNA)*100
NC_004552	330816	1144377	86.7238680959
NC_008700	1244887	4306142	86.7287005398
NC_006360	258131	892758	86.7416477926
NC_004463	2633751	9105828	86.7713842168
NC_004369	910390	3147090	86.7839813923
NC_005966	1041259	3598621	86.8048344074
NC_006576	780686	2696255	86.8633716025
NC_009636	1095213	3781904	86.8779059437
NC_007963	1071330	3696649	86.9433370601
NC_004432	393757	1358633	86.9455548334
NC_008309	582003	2007700	86.9656323156
NC_009441	1768022	6096872	86.9965123099
NC_007626	1440580	4967148	87.0064672927
NC_009675	1531045	5277990	87.0243217589
NC_000922	356964	1230230	87.0481129545
NC_008027	1709165	5888780	87.0722798271
NC_008541	1363897	4698945	87.076801282
NC_006814	578764	1993560	87.0950460483
NC_009725	1138327	3918589	87.1482311618
NC_009436	1312828	4518712	87.1594383532
NC_008525	532512	1832387	87.1833297224
NC_009648	1544895	5315120	87.1981253481
NC_009943	1146790	3944167	87.2267832473
NC_009664	1384568	4761183	87.2410071194
NC_009655	674572	2319663	87.2418105561
NC_008570	1380036	4744448	87.2621641127
NC_009785	639106	2196662	87.2832506776

Table S4. *(Continued)*

NC Number	Amino acid	DNA	(Amino*3/DNA)*100
NC_002944	1405986	4829781	87.3322827681
NC_007947	865053	2971517	87.3344826902
NC_009654	1485015	5100344	87.3479318258
NC_008340	953862	3275944	87.3514931879
NC_010002	1971489	6767514	87.3949725113
NC_008530	551955	1894360	87.4102599295
NC_000964	1228401	4215606	87.4181078592
NC_009434	1330944	4567418	87.4198945663
NC_007005	1776757	6093698	87.4718602727
NC_007514	750078	2572079	87.4869706568
NC_008254	1286900	4412446	87.4956883325
NC_010170	1542644	5287950	87.5184523303
NC_008555	821310	2814130	87.5556566328
NC_008343	790774	2708355	87.5927269505
NC_009792	1378562	4720462	87.6118905311
NC_002932	629979	2154946	87.7022904518
NC_002936	429956	1469720	87.7628391803
NC_008044	936549	3200938	87.7757394864
NC_007513	654745	2234828	87.8919988473
NC_008025	722888	2467205	87.8996273111
NC_007164	721591	2462499	87.9095991511
NC_004129	2074323	7074893	87.9584892662
NC_009439	1487854	5072807	87.9899826664
NC_009719	1148880	3914745	88.0425161792
NC_007908	1383329	4712337	88.0664307328
NC_007643	1277856	4352825	88.0708045924
NC_008009	1658947	5650368	88.0799445275

Table S4. *(Continued)*

NC Number	Amino acid	DNA	(Amino*3/DNA)*100
NC_009480	968980	3297891	88.1454238482
NC_008347	990354	3368780	88.1940049513
NC_007512	696443	2364842	88.3496233575
NC_009952	1116273	3789584	88.3690399791
NC_002942	1001232	3397754	88.4023975838
NC_003212	887344	3011208	88.4041221995
NC_008571	1120032	3798465	88.4593118536
NC_002663	665692	2257487	88.4645625866
NC_003911	1211874	4109437	88.4700750979
NC_009009	704421	2388435	88.4789830998
NC_008095	2696166	9139763	88.4978964991
NC_002947	1824445	6181873	88.5384575193
NC_008255	1309277	4433218	88.5999966616
NC_003888	2560626	8667507	88.6284602943
NC_007802	1276378	4317977	88.678888285
NC_008148	953835	3225748	88.7082623937
NC_008702	1294297	4376040	88.7307017303
NC_009482	658535	2224914	88.7946680186
NC_010003	642534	2169548	88.8480918606
NC_003361	347592	1173390	88.868662593
NC_008048	991489	3345170	88.9182612543
NC_008601	566237	1910031	88.9363052223
NC_009455	397884	1341892	88.9529112626
NC_003210	873580	2944528	89.00373846
NC_007503	712557	2401520	89.0132499417
NC_002516	1858863	6264404	89.020264338
NC_005125	1384349	4659019	89.1399455551

Table S4. *(Continued)*

NC Number	Amino acid	DNA	(Amino*3/DNA)*100
NC_008209	1228326	4133097	89.1577913608
NC_008740	1286430	4326849	89.1940070014
NC_007356	414954	1395502	89.2053182296
NC_008358	1102631	3705021	89.2813563
NC_004663	1864384	6260361	89.3423238692
NC_008789	798043	2678452	89.3848013703
NC_000962	1315575	4411532	89.4638189182
NC_000117	311221	1042519	89.5583677612
NC_004113	774371	2593857	89.5621077029
NC_007517	1194964	3997420	89.6801436927
NC_000919	340208	1138011	89.6848975977
NC_009718	582670	1948941	89.690247165
NC_007520	726099	2427734	89.7255218241
NC_009663	766826	2562277	89.7825644924
NC_007722	913870	3052398	89.8182347125
NC_007298	1347971	4501104	89.8426919262
NC_006347	1580916	5277274	89.8711721241
NC_002620	321863	1072950	89.9938487348
NC_008726	1949293	6491865	90.0801079505
NC_007404	874520	2909809	90.162618921
NC_009484	1018738	3389227	90.1743671935
NC_010163	450261	1496992	90.2331475385
NC_009481	711971	2366980	90.2378980811
NC_009379	649616	2159490	90.2457524693
NC_002945	1307748	4345492	90.2830795684
NC_002696	1209123	4016947	90.3016395287
NC_006908	234075	777079	90.3672599568

Table S4. *(Continued)*

NC Number	Amino acid	DNA	(Amino*3/DNA)*100
NC_001318	274481	910724	90.4163061476
NC_004917	542536	1799146	90.4655875621
NC_008599	534994	1773615	90.4921304793
NC_009337	593682	1966858	90.5528512989
NC_009511	1625243	5382261	90.5888621901
NC_002939	1151872	3814128	90.600420332
NC_009616	578809	1915238	90.6637712911
NC_009338	1701087	5619607	90.8117062279
NC_002927	1617520	5339179	90.8858833914
NC_008596	2118636	6988209	90.9518876725
NC_009077	1841159	6048425	91.3209141223
NC_002163	499747	1641481	91.334654498
NC_002967	866832	2843201	91.4636707007
NC_000918	473565	1551335	91.5788659445
NC_008146	1747425	5705448	91.8819170729
NC_008705	1758291	5737227	91.9411590303
NC_006512	871714	2839318	92.1045828611
NC_005363	1162663	3782950	92.2028839927
NC_007575	677430	2201561	92.3113191049
NC_006156	278306	904246	92.3330598089
NC_008277	279188	905394	92.5082339843
NC_006055	244754	793224	92.5667907174
NC_005835	586358	1894877	92.8331495923
NC_009850	724838	2341251	92.8782945528
NC_005090	655558	2110355	93.1916194195
NC_009828	664125	2135342	93.304725894
NC_009486	569389	1823511	93.674620005

Table S4. *(Continued)*

NC Number	Amino acid	DNA	(Amino*3/DNA)*100
NC_009662	590353	1877931	94.3090560835
NC_000853	584974	1860725	94.3138830295
NC_007205	417754	1308759	95.7595707078
NC_005027	2306089	7145576	96.8188848597
