# Supplementary material for: A Preliminary Metagenome Analysis Based on a Combination of Protein Domains
Source: Proteomes. 2019 Apr 29;7(2):19. doi: 10.3390/proteomes7020019 (PMC6630717; doi:10.3390/proteomes7020019)
Supplement: Supplementary file 1 [file proteomes-07-00019-s001.zip › supplementary/Figure S7.pptx]

## Slide 1
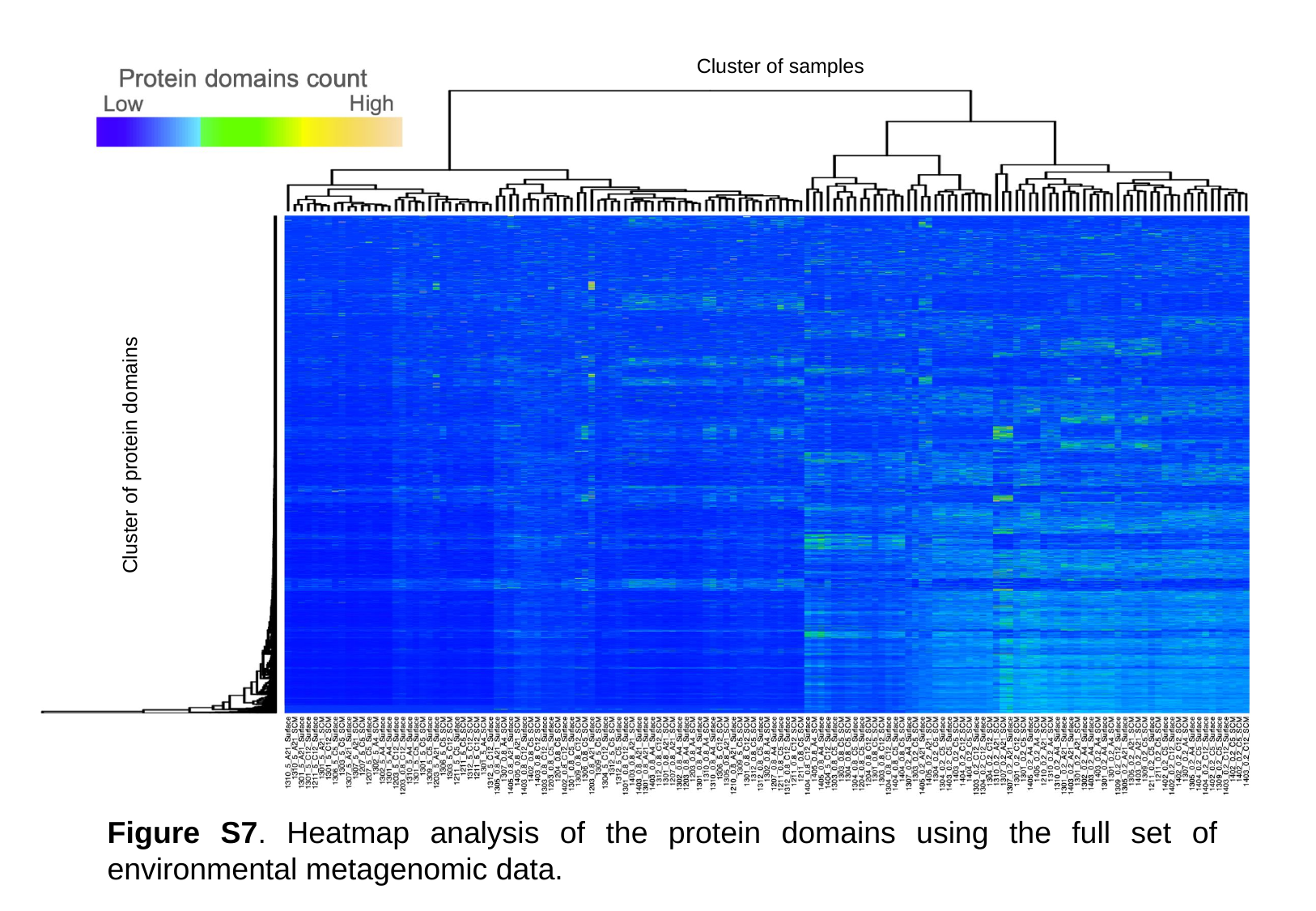

Cluster of samples
Cluster of protein domains
Figure S7. Heatmap analysis of the protein domains using the full set of environmental metagenomic data.
