# Supplementary material for: A Preliminary Metagenome Analysis Based on a Combination of Protein Domains
Source: Proteomes. 2019 Apr 29;7(2):19. doi: 10.3390/proteomes7020019 (PMC6630717; doi:10.3390/proteomes7020019)
Supplement: Supplementary file 1 [file proteomes-07-00019-s001.zip › supplementary/Figure S6.pptx]

## Slide 1
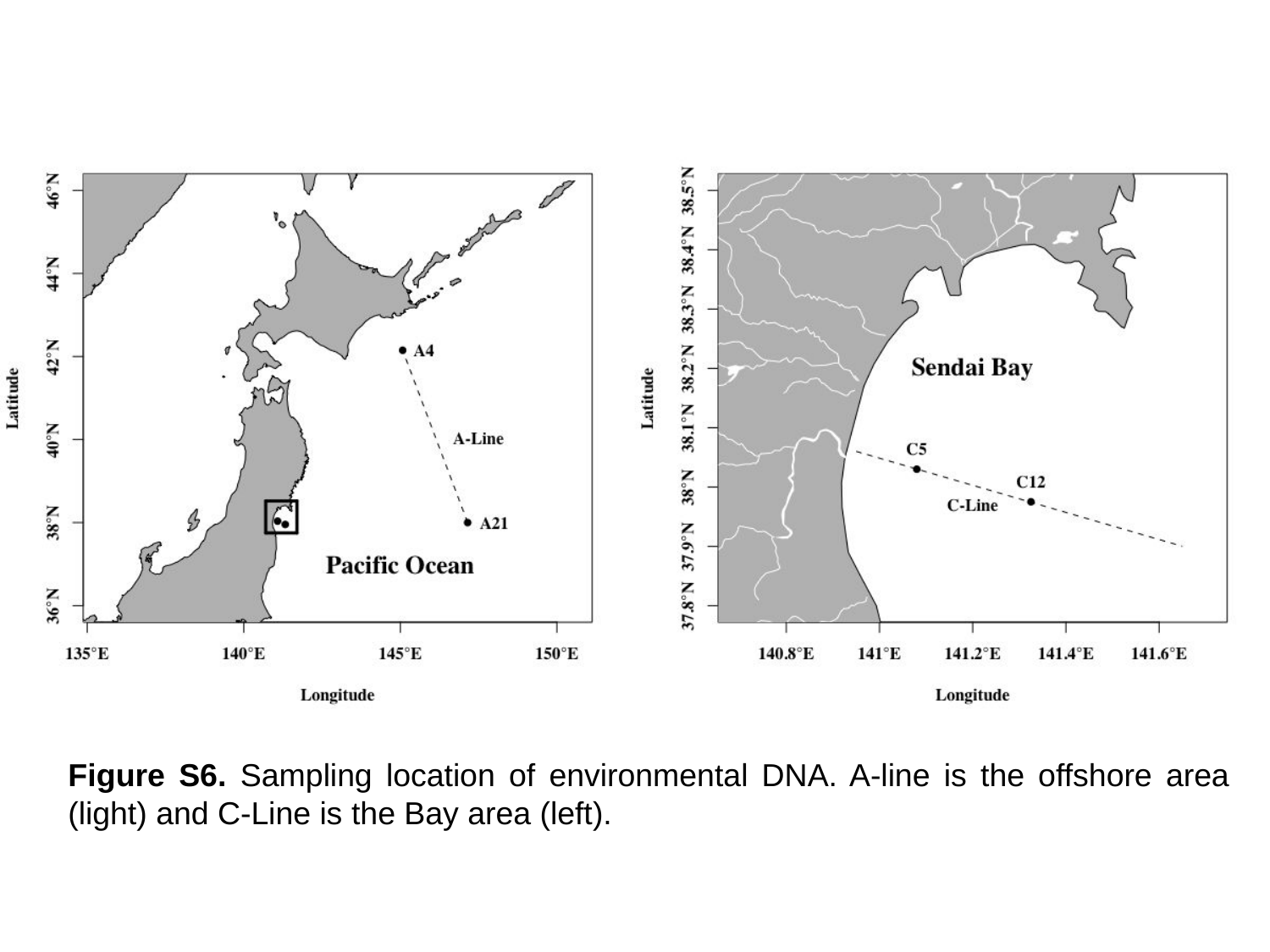

Figure S6. Sampling location of environmental DNA. A-line is the offshore area (light) and C-Line is the Bay area (left).
