# Supplementary figures and images for: A Preliminary Metagenome Analysis Based on a Combination of Protein Domains
Source: Proteomes. 2019 Apr 29;7(2):19. doi: 10.3390/proteomes7020019 (PMC6630717; doi:10.3390/proteomes7020019)

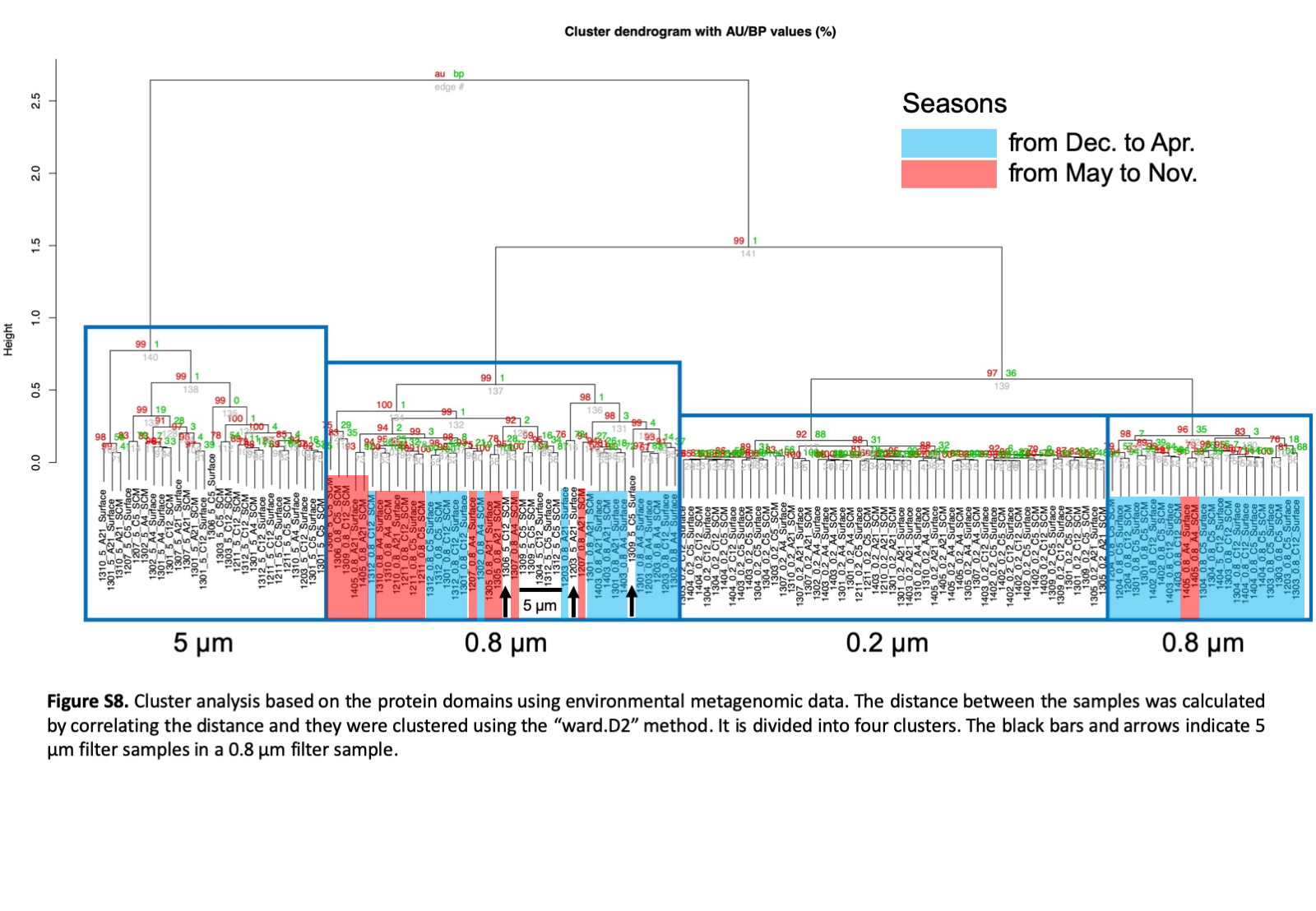

Supplement: Supplementary file 1 [file proteomes-07-00019-s001.zip › supplementary/Figure S8.tiff]

## Slide 1
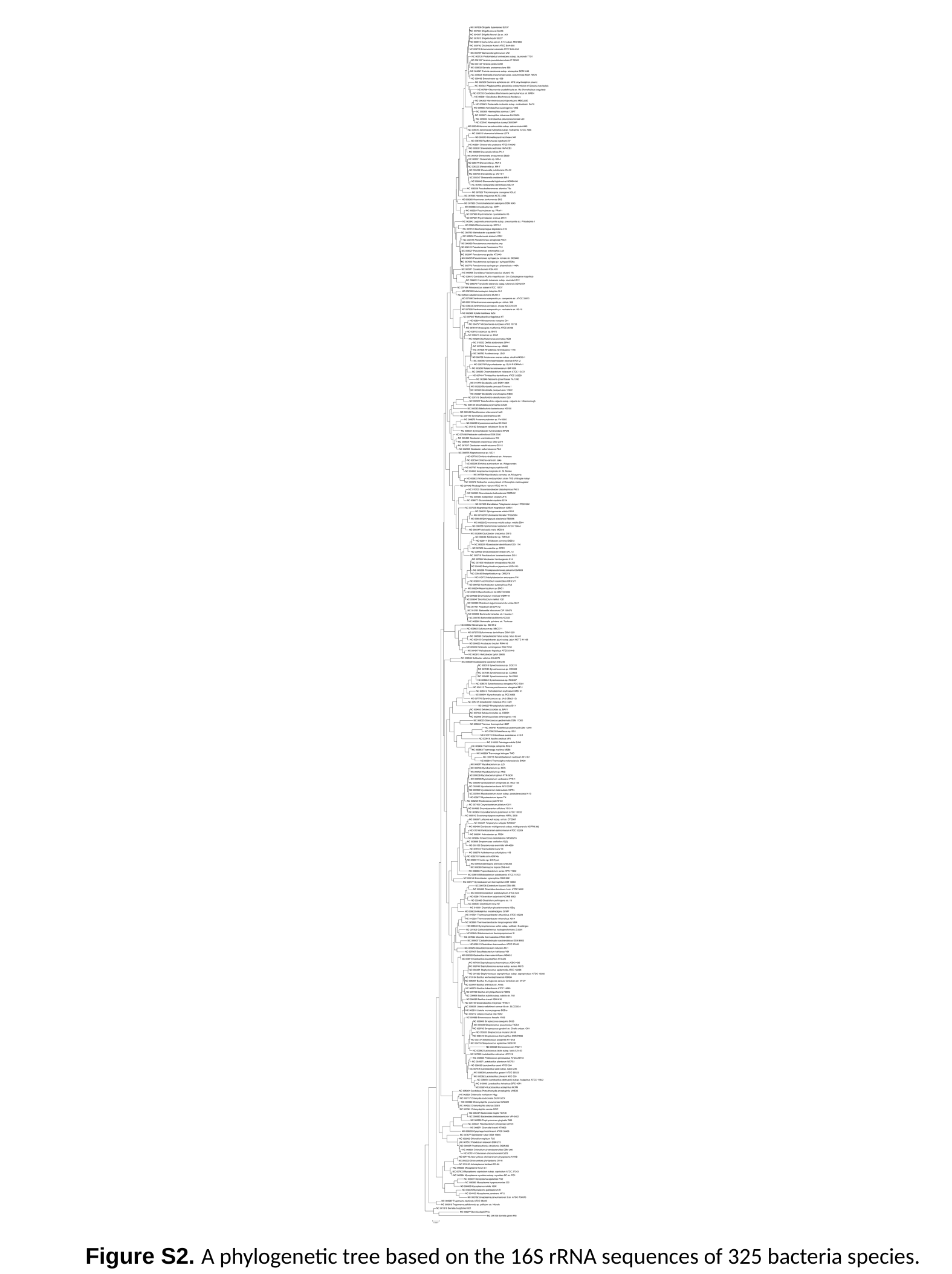

Figure S2. A phylogenetic tree based on the 16S rRNA sequences of 325 bacteria species.

Supplement: Supplementary file 1 [file proteomes-07-00019-s001.zip › supplementary/Figure S2.pptx]
